# Stimuli-responsive bioengineered platforms for precision cancer therapy

**DOI:** 10.3389/fbioe.2026.1872451

**Published:** 2026-08-26

**Authors:** Hossein Omidian, Renae L. Wilson

**Affiliations:** Barry and Judy Silverman College of Pharmacy, Nova Southeastern University, Fort Lauderdale, FL, United States

**Keywords:** bioengineered nanomedicine, controlled drug delivery, multifunctional biomaterials, precision oncology, stimuli-responsive biomaterials, stimuli-responsive nanomedicine, translational cancer bioengineering, tumor microenvironment

## Abstract

Stimuli-responsive bioengineered platforms are redefining cancer therapy by shifting therapeutic design from systemic drug exposure toward context-dependent activation within malignant tissue. These systems are engineered to sense and respond to tumor-associated or externally applied cues, including acidic pH, redox imbalance, hypoxia, enzymatic activity, reactive oxygen species, temperature variation, light, ultrasound, and magnetic fields. Across the reviewed evidence, their principal value lies not merely in drug encapsulation but in the coordinated control of localization, release, intracellular access, multimodal therapy, microenvironment modulation, and safety. The field encompasses diverse architectures, including biomacromolecular nanoparticles, hydrogels, nanogels, polymeric micelles, prodrug assemblies, lipid-based systems, mesoporous silica, metal–organic frameworks, carbon-based materials, magnetic nanocomposites, and hybrid inorganic–organic constructs. These platforms have been validated in multiple cancer models through assays of uptake, cytotoxicity, apoptosis, spheroid penetration, tumor suppression, metastasis, recurrence, immune activation, stromal remodeling, and systemic tolerability. Collectively, the evidence supports a conceptual transition from passive nanocarriers to programmable therapeutic systems capable of aligning therapeutic action with the spatial, temporal, and biological heterogeneity of tumors. However, translation remains constrained by formulation complexity, incomplete standardization, limited long-term safety and biodistribution data, insufficient penetration into protected tumor niches, and the need for clinically relevant models that capture patient-level heterogeneity. Future progress will depend on rationally simplified architectures, quantitative stimulus–response validation, scalable manufacturing, integrated safety assessment, and biomarker-guided selection of platforms matched to defined tumor microenvironments. Stimuli-responsive bioengineering therefore represents a promising, although still maturing, foundation for safer, more selective, and more mechanistically coordinated precision cancer therapy.

## Graphical Abstract

**Figure:**
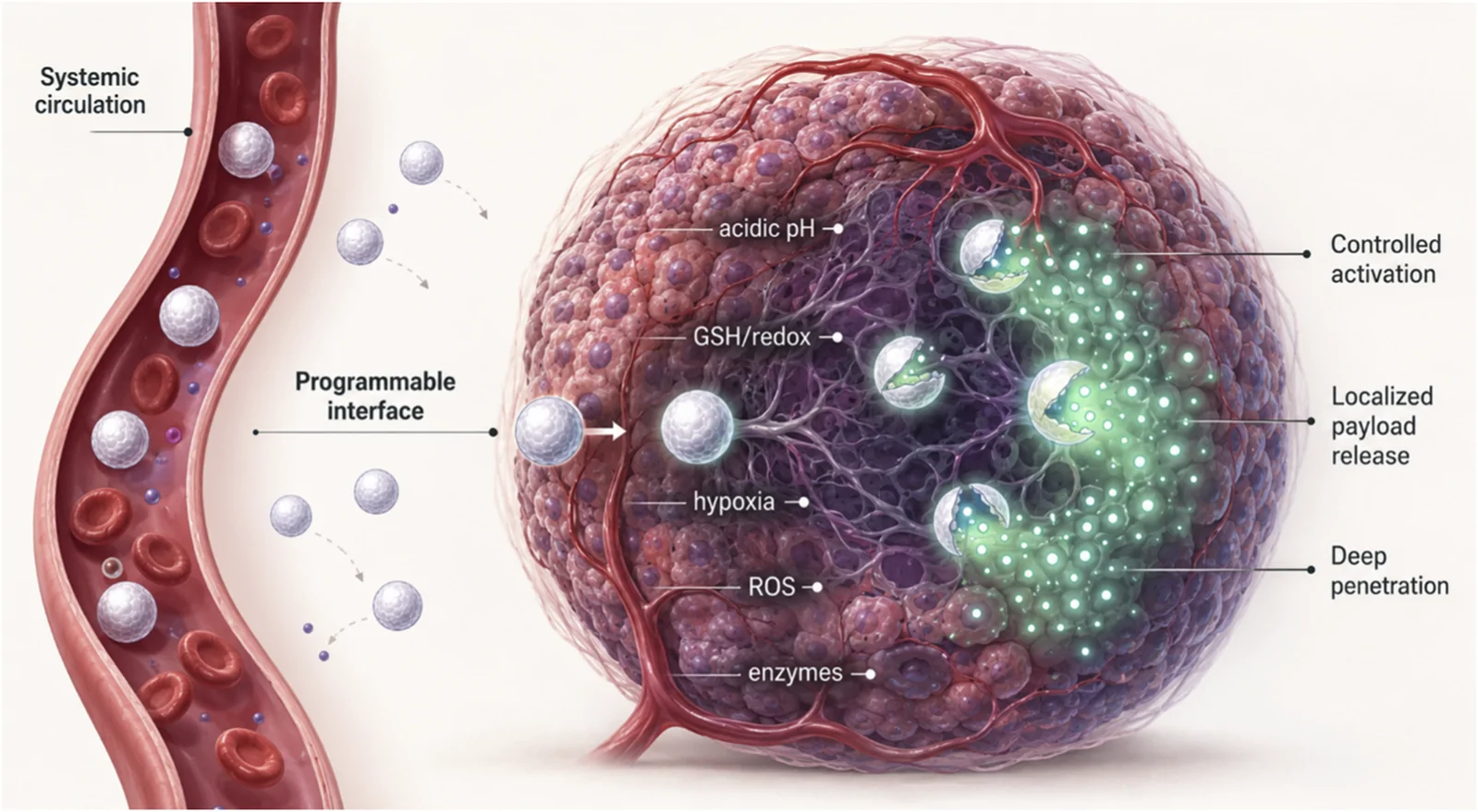
(Prepared with the assistance of Gemini’s NotebookLM Pro and ChatGPT Plus).

## Introduction

Cancer therapy increasingly demands strategies that do more than intensify cytotoxicity. Tumors are spatially heterogeneous, biologically adaptive, and protected by microenvironmental barriers that limit drug penetration, impair immune activity, promote resistance, and create regions of therapeutic escape. Conventional systemic administration often fails to reconcile three competing requirements: adequate tumor exposure, controlled intracellular activation, and reduced injury to normal tissues. Stimuli-responsive bioengineered platforms have emerged from this unmet need as therapeutic systems designed to couple drug release, carrier transformation, or biological activation to disease-relevant conditions rather than to systemic circulation alone (Abd Al-Jabbar et al., 2022; Augustine et al., 2020; Chang et al., 2022; Christodoulou et al., 2025; Gebrie et al., 2022; Hosseini et al., 2024; Kazemi et al., 2025a; Lin et al., 2021; Miao et al., 2020; Ozkahraman et al., 2026; Sasikala et al., 2025; Tao and Yin, 2020; Wang et al., 2024a; Yang et al., 2020).

The defining principle of these platforms is stimulus–material coupling. Instead of functioning as inert containers, responsive systems are engineered to undergo programmed changes such as degradation, deshielding, swelling, charge conversion, disassembly, photothermal conversion, photodynamic activation, or enzyme-mediated remodeling after exposure to biological or externally controlled triggers. Acidic pH has been used to promote release, matrix destabilization, framework dissolution, and charge conversion; redox and glutathione gradients have enabled intracellular activation through disulfide cleavage, coordination disruption, and prodrug release; and hypoxia, reactive oxygen species, enzymes, temperature, light, and ultrasound have expanded the repertoire of tumor-adapted or temporally controlled activation mechanisms (Augustine et al., 2020; Chen et al., 2026; Du et al., 2020; Gupta et al., 2022; Hu et al., 2022; Li et al., 2021; Mamnoon et al., 2020; Rashidzadeh et al., 2023; Tao and Yin, 2020; Wang L. et al., 2025; Xiao et al., 2021; Zhang D. et al., 2024; Zhang Y. P. et al., 2025).

This mechanistic diversity is supported by broad architectural diversity. The reviewed platforms include biomacromolecular carriers, protein-derived nanoparticles, hydrogels, nanogels, polymeric micelles, vesicles, prodrug nanoparticles, lipid-based systems, mesoporous silica, organosilica, metal–organic frameworks, covalent organic frameworks, carbon-based materials, magnetic nanocomposites, and hybrid inorganic–organic constructs. Although these materials differ substantially in composition, their shared architecture integrates four functional elements: a structural carrier matrix, a therapeutic or theranostic cargo, a stimulus-responsive module, and a precision-delivery interface that supports localization, penetration, intracellular access, or signal-triggered release (Abd Al-Jabbar et al., 2022; Arvejeh et al., 2025; Augustine et al., 2020; Curcio et al., 2020; Gao et al., 2025; Guo et al., 2020; Li et al., 2024; Liu et al., 2025; Mahani et al., 2025; Ozkahraman et al., 2026; Rashidzadeh et al., 2023; Tao and Yin, 2020; Yan et al., 2020; Zhang F. et al., 2023; Zhang and Liang, 2024; Zhou et al., 2023).

The therapeutic rationale for these systems extends beyond controlled release. Many platforms were designed to coordinate multiple anticancer mechanisms within a single construct, including dual-drug chemotherapy, gene–drug co-delivery, chemoimmunotherapy, chemo-photothermal therapy, chemo-photodynamic therapy, chemodynamic therapy, ferroptosis-associated strategies, radiosensitization, sonodynamic activation, and protein–photosensitizer delivery. This multifunctional logic is especially relevant where tumor heterogeneity, multidrug resistance, immune suppression, recurrence, metastasis, or poor monotherapy response limits durable efficacy (Arvejeh et al., 2025; Gao et al., 2025; Li et al., 2025; Liu et al., 2025; Phung et al., 2023; Song et al., 2023; Wang et al., 2026; Yan et al., 2020; Yan et al., 2023; Yang et al., 2022; Zhang F. et al., 2023; Zhang G. Z. et al., 2023; Zhang L. et al., 2024; Zhang L. et al., 2025; Zhang N. X. et al., 2021; Zhang Y. P. et al., 2025; Zhang and Liang, 2024; Zhou et al., 2023). In this context, precision is not achieved simply by adding more agents; it is achieved by synchronizing complementary mechanisms within tumor-relevant biological windows (Puzzo et al., 2025; Puzzo et al., 2024).

A second major rationale is the need to overcome tumor transport barriers. Passive accumulation alone is insufficient when dense stroma, hypoxia, high interstitial pressure, vascular distance, extracellular matrix architecture, and resistant cellular niches prevent adequate exposure. Spatial mapping studies showing that liposomal nanoparticles may fail to diffuse beyond approximately 100 μm from nearby blood vessels underscore the importance of penetration as a distinct design requirement rather than a secondary pharmacokinetic outcome (Samson et al., 2020). Accordingly, responsive platforms have incorporated charge reversal, size transition, matrix degradation, transcytosis-oriented transport, macrophage-mediated delivery, stromal remodeling, and tumor microenvironment modulation to improve access to poorly reached compartments (Jia et al., 2022; Jiang et al., 2025; Qian et al., 2020; Shi et al., 2020; Tan et al., 2024; Wang et al., 2020; Wang L. J. et al., 2025; Wang et al., 2024a; Wang et al., 2024b).

Preclinical validation supports the biological plausibility of this design logic. Across breast, colorectal, lung, prostate, hepatic, cervical, gastric, ovarian, glioblastoma, and other solid tumor models, responsive platforms have improved cellular uptake, cytotoxicity, apoptosis, tumor suppression, immune activation, metastasis control, recurrence reduction, and survival-related outcomes compared with free drugs, monotherapies, nonresponsive carriers, or nontargeted controls (Abd Al-Jabbar et al., 2022; Arvejeh et al., 2025; Ayyanaar et al., 2020; Bhattacharya et al., 2024; Chen et al., 2026; Fu et al., 2025; Gupta et al., 2024; Li et al., 2024; Rahmani et al., 2021; Song et al., 2023; Yan et al., 2020; Yan et al., 2023; Zhang et al., 2022; Zhang Y. P. et al., 2025). Importantly, the most compelling evidence does not rely solely on short-term viability reduction. It links therapeutic benefit to stimulus-linked uptake, spheroid or tissue penetration, tumor microenvironment remodeling, immune activation, and reduced systemic or normal-cell toxicity (Du et al., 2020; González-Urías et al., 2021; Mamnoon et al., 2020; Migasova et al., 2025; Rashidzadeh et al., 2023; Samson et al., 2020; Wang et al., 2020; Wang L. J. et al., 2025; Xiao et al., 2021).

Taken together, the reviewed literature positions stimuli-responsive bioengineering as a precision-therapy framework rather than a single material class. Its translational significance lies in engineering systems that can sense tumor-associated cues, protect therapeutic cargo during circulation, release or activate payloads in defined biological compartments, coordinate multiple therapeutic modalities, and reduce nonspecific toxicity. The field is therefore moving from the question of whether nanocarriers can deliver drugs toward a more demanding question: whether programmable biomaterials can be designed, validated, manufactured, and clinically matched to the biological complexity of individual tumors (González-Urías et al., 2021; Mahani et al., 2025; Namazi et al., 2023; Perera et al., 2022; Tang et al., 2021; Wang et al., 2024b; Zhang G. Z. et al., 2023).

## Therapeutic bioengineering objective

### Precision-oriented rationale for stimuli-responsive delivery

The therapeutic objective of stimuli-responsive bioengineered platforms is to improve the spatial, temporal, and biological control of cancer therapy. Across the reviewed studies, these systems were primarily designed to address limitations of conventional anticancer treatment, including poor tumor selectivity, premature systemic exposure, inadequate intracellular release, insufficient tumor accumulation, and treatment-associated toxicity. Rather than functioning only as passive carriers, stimuli-responsive platforms were developed to link therapeutic activation to tumor- or cell-associated conditions, including acidity, redox imbalance, hypoxia, reactive oxygen species, enzyme activity, temperature changes, and charge-conversion behavior (Abd Al-Jabbar et al., 2022; Augustine et al., 2020; Chang et al., 2022; Christodoulou et al., 2025; Gebrie et al., 2022; Hosseini et al., 2024; Kazemi et al., 2025a; Lin et al., 2021; Miao et al., 2020; Ozkahraman et al., 2026; Sasikala et al., 2025; Tao and Yin, 2020; Wang et al., 2024a; Yang et al., 2020). This therapeutic logic directly supports precision cancer therapy: the objective is not merely to transport a therapeutic agent, but to improve when, where, and under which biological conditions treatment becomes active.

**FIGURE 1:**
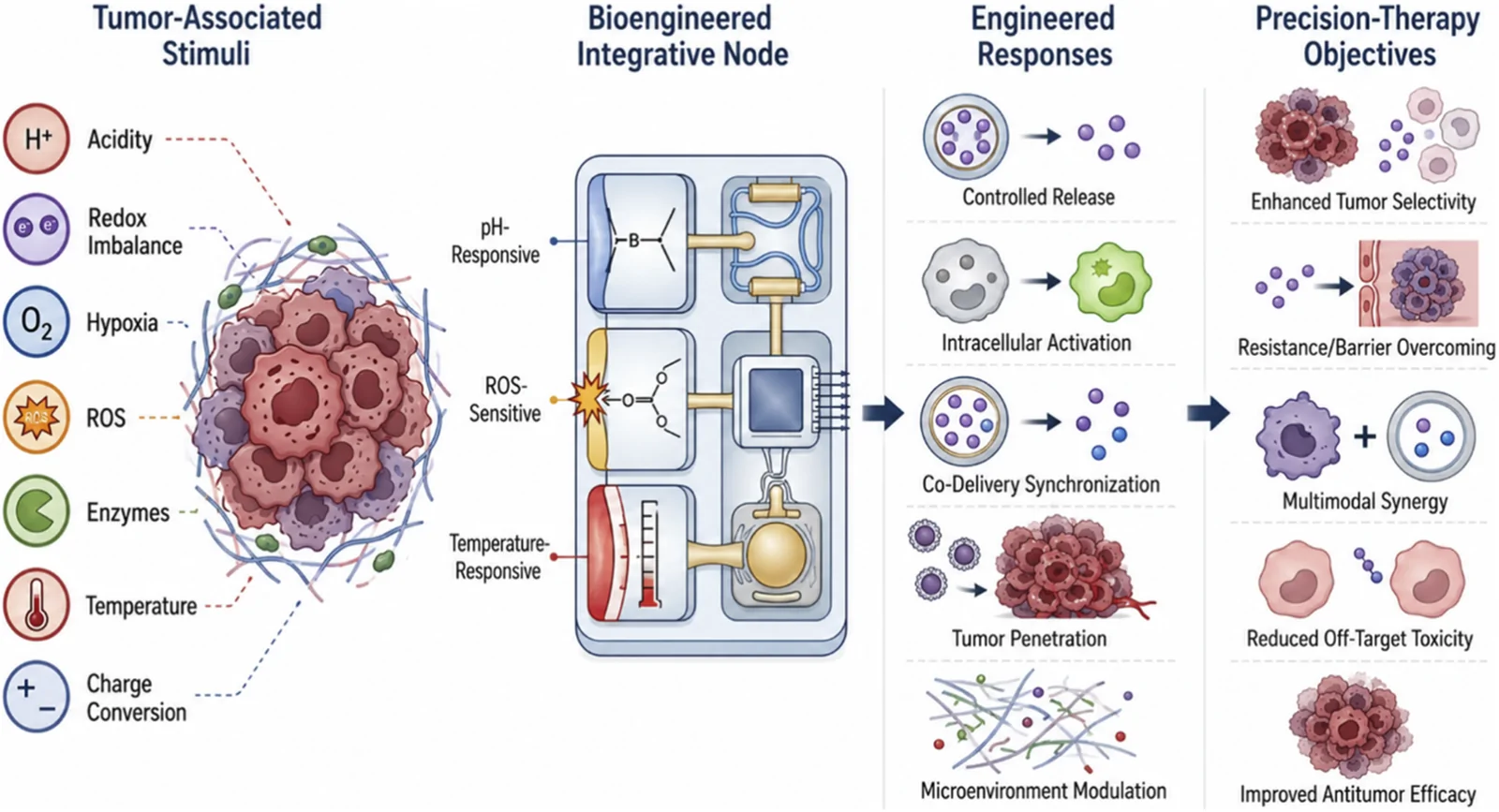
Therapeutic bioengineering objectives of stimuli-responsive platforms in precision cancer therapy. Tumor-associated stimuli, including acidity, redox imbalance, hypoxia, ROS, enzyme activity, temperature, and charge conversion, can be exploited as biological inputs for responsive therapeutic systems. The bioengineered platform functions as an integrative control node that links tumor-specific cues to programmed therapeutic responses. These responses include controlled release, intracellular activation, synchronized co-delivery, improved penetration, and tumor microenvironment modulation. Together, these mechanisms support enhanced tumor selectivity, barrier overcoming, multimodal synergy, reduced off-target toxicity, and improved antitumor efficacy (Prepared with the assistance of Gemini’s NotebookLM Pro and ChatGPT Plus).

The reviewed studies applied this principle across breast, colorectal, pancreatic, lung, prostate, cervical, gastric, liver, ovarian, glioblastoma, and other solid tumor settings. Although the disease contexts differed, the shared objective was consistent: to increase tumor-localized therapeutic action while reducing exposure of nonmalignant tissues (Bilchi et al., 2025; Gupta et al., 2022; Huang et al., 2022; Kv et al., 2020; Mahani et al., 2025; Migasova et al., 2025; Raj and Sundaramoorthy, 2025; Sujatha et al., 2025; Wei et al., 2024; Xiao et al., 2021; Zhang and Liang, 2024). In this section, stimuli-responsiveness is therefore interpreted as a therapeutic design principle rather than as a material feature alone. The conceptual relationship between tumor-associated stimuli, engineered responses, and precision-therapy objectives is summarized in Figure 1, and the major objective patterns connecting responsive bioengineering to cancer therapy are organized in Table 1.

**TABLE 1:** Therapeutic bioengineering objectives linking stimuli-responsive design to precision cancer therapy.

Therapeutic bioengineering objective	Stimuli-responsive precision basis	Therapeutic limitation or objective-linked constraint	Pattern revealed for precision cancer therapy	Representative references
Tumor-selective activation and intracellular therapeutic release	Therapeutic action is coupled to tumor-associated acidity, redox imbalance, hypoxia, reactive oxygen species, enzyme activity, temperature, or charge conversion	Conventional therapy is limited by poor tumor selectivity, premature systemic exposure, and inadequate intracellular release	Stimuli-responsiveness functions as a biological control mechanism that restricts therapeutic activation to malignant tissues or intracellular tumor compartments	(Abd Al-Jabbar et al., 2022; Augustine et al., 2020; Chang et al., 2022; Christodoulou et al., 2025; Gebrie et al., 2022; Hosseini et al., 2024; Kazemi et al., 2025a; Lin et al., 2021; Miao et al., 2020; Ozkahraman et al., 2026; Sasikala et al., 2025; Tao and Yin, 2020; Wang et al., 2024a; Yang et al., 2020)
Coordinated co-delivery and multimodal therapeutic action	Responsive systems synchronize the release or activation of multiple therapeutic functions within tumor-relevant environments	Single-agent therapy and conventional combinations are limited by resistance, poor therapeutic coordination, mismatched biodistribution, and dose-limiting toxicity	Precision is achieved by aligning complementary therapeutic mechanisms at the tumor site rather than merely combining agents systemically	(Abd Al-Jabbar et al., 2022; Arvejeh et al., 2025; Curcio et al., 2020; Gao et al., 2025; Gupta et al., 2022; Jin et al., 2021a; Li et al., 2025; Liu et al., 2025; Qiu et al., 2021; Sauraj et al., 2020; Wang et al., 2026; Yan et al., 2020; Zhang et al., 2021a; Zhang and Liang, 2024; Puzzo et al., 2025; Puzzo et al., 2024)
Enhanced access to resistant or poorly penetrated tumor regions	Responsive changes in charge, size, tissue interaction, stromal remodeling, transcytosis, or cell-mediated transport are used to improve tumor access	Solid tumors contain poorly accessible regions; standard liposomal nanoparticles may fail to diffuse beyond approximately 100 μm from nearby blood vessels	Precision cancer therapy requires physical delivery into protected tumor compartments, not only molecular or cellular targeting	(Jia et al., 2022; Jiang et al., 2025; Qian et al., 2020; Samson et al., 2020; Shi et al., 2020; Tan et al., 2024; Wang et al., 2020; Wang et al., 2025b; Wang et al., 2024b; Zhang et al., 2021a)
Reversal of resistance, recurrence, and treatment-failure mechanisms	Stimuli-responsive platforms integrate cytotoxic delivery with gene modulation, chemosensitization, immune stimulation, radiosensitization, or anti-metastatic intervention	Multidrug resistance, chemoresistance, recurrence, metastasis, and therapy-induced adaptive survival reduce durable treatment efficacy	Responsive bioengineering is used to couple delivery with biological sensitization in tumors where drug exposure alone is insufficient	(Hu et al., 2022; Karimifard et al., 2022; Mathes et al., 2024; Rahmani et al., 2021; Zhang et al., 2023a; Zhang et al., 2025a; Zhang et al., 2022; Zhou et al., 2023; Puzzo et al., 2025; Puzzo et al., 2024)
Tumor microenvironment remodeling and immune activation	Stimuli-triggered delivery is used to modulate immunosuppression, hypoxia, stromal barriers, macrophage polarization, or antitumor immune recruitment	The tumor microenvironment can suppress immune responses, restrict therapy, and support invasion, metastasis, or recurrence	Precision platforms increasingly aim to recondition the disease niche, not only deliver cytotoxic agents	(Gao et al., 2025; Jiang et al., 2025; Li et al., 2025; Phung et al., 2023; Song et al., 2023; Tang et al., 2021; Yan et al., 2020; Yan et al., 2023; Zhang et al., 2023b; Zhang et al., 2024b; Zhang et al., 2025a)
Improved therapeutic index, localized precision, and safety	Controlled activation, localized delivery, safer clearance, or reduced premature release limits exposure outside the intended therapeutic site	Systemic toxicity, off-target exposure, rapid clearance, premature leakage, and long-term material retention constrain clinical usefulness	Safety and efficacy are treated as linked design objectives; precision is defined by selective benefit as well as reduced harm	(Gupta et al., 2024; Jia et al., 2020; Li et al., 2024; Miao et al., 2020; Perera et al., 2022; Raj and Sundaramoorthy, 2025; Vaghasiya et al., 2021; Wang et al., 2025a; Xiao et al., 2021; Yang et al., 2020; Zhang et al., 2024a; Zhang et al., 2025b)

### Coordinated co-delivery and multimodal therapy

A major therapeutic objective across the literature was to coordinate multiple anticancer mechanisms within a single responsive system. Co-delivery strategies were designed to address limitations of single-agent treatment, including drug resistance, inadequate therapeutic synergy, mismatched biodistribution, and dose-limiting toxicity. In this context, multidrug therapy is most relevant when each component contributes a distinct therapeutic function, such as direct cytotoxicity, gene modulation, immune stimulation, radiosensitization, microenvironment remodeling, or suppression of resistance-associated survival pathways (Abd Al-Jabbar et al., 2022; Arvejeh et al., 2025; Curcio et al., 2020; Gao et al., 2025; Gupta et al., 2022; Jin M. J. et al., 2021; Li et al., 2025; Liu et al., 2025; Qiu et al., 2021; Sauraj et al., 2020; Wang et al., 2026; Yan et al., 2020; Zhang B. B. et al., 2021; Zhang and Liang, 2024; Zhou et al., 2023; Puzzo et al., 2025; Puzzo et al., 2024). These platforms aimed to synchronize chemotherapy with complementary interventions such as gene modulation, immune activation, photothermal or photodynamic therapy, sonodynamic therapy, ferroptosis or chemodynamic-enhanced therapy, radiosensitization, extracellular matrix modulation, or anti-metastatic treatment (Abd Al-Jabbar et al., 2022; Arvejeh et al., 2025; Curcio et al., 2020; Gao et al., 2025; Gupta et al., 2022; Jin M. J. et al., 2021; Li et al., 2025; Liu et al., 2025; Qiu et al., 2021; Sauraj et al., 2020; Wang et al., 2026; Yan et al., 2020; Zhang B. B. et al., 2021; Zhang and Liang, 2024). The central therapeutic purpose of these approaches was not simply to combine agents but to align multiple therapeutic actions with tumor-relevant biological conditions.

This objective was particularly important in settings where resistance, recurrence, immune suppression, or heterogeneous tumor biology limited the effectiveness of conventional treatment. Responsive co-delivery systems were used to strengthen chemotherapy-based treatment, enhance immunogenic or immune-mediated activity, improve intracellular therapeutic access, and support sequential or coordinated release in tumor-relevant compartments (Jiang et al., 2025; Kuang et al., 2025; Lin et al., 2024; Mathes et al., 2024; Phung et al., 2023; Song et al., 2023; Wang L. et al., 2025; Yang et al., 2022; Zhang F. et al., 2023; Zhang L. et al., 2024; Zhang Y. P. et al., 2025; Zhou et al., 2023). In the context of precision cancer therapy, co-delivery therefore represents a strategy for matching therapeutic timing, location, and mechanism to the biological constraints of the tumor microenvironment.

### Overcoming tumor barriers and treatment resistance

Another central therapeutic objective was to overcome physical and biological barriers that restrict effective treatment in solid tumors. These barriers included multidrug resistance, chemoresistance, stromal density, dense extracellular matrix, hypoxia, immunosuppression, poor vascular-to-tumor transport, and inadequate penetration into deep tumor regions (Hu et al., 2022; Jia et al., 2022; Jiang et al., 2025; Karimifard et al., 2022; Li Y. C. et al., 2020; Mathes et al., 2024; Tang et al., 2021; Wang L. J. et al., 2025; Zhang F. et al., 2023; Zhang L. et al., 2025; Zhang et al., 2022; Zhou et al., 2023). One study directly illustrated the magnitude of the penetration challenge by showing that standard liposomal nanoparticles failed to diffuse more than approximately 100 μm from the nearest blood vessel, leaving hypoxic and clonogenic tumor cells beyond effective liposomal reach (Samson et al., 2020). This finding clarifies why precision cancer therapy requires more than molecular recognition or tumor-selective activation; it also requires physical access to poorly reached tumor compartments.

In response to these barriers, multiple platforms were designed to enhance tumor penetration, promote intracellular or nuclear delivery, remodel the tumor microenvironment, improve immune activation, support macrophage repolarization, or increase therapeutic access within resistant tumor compartments (Jia et al., 2022; Jiang et al., 2025; Phung et al., 2023; Qian et al., 2020; Shi et al., 2020; Tan et al., 2024; Wang et al., 2020; Wang L. J. et al., 2025; Wang et al., 2024b; Zhang G. Z. et al., 2023; Zhang L. et al., 2024; Zhang L. et al., 2025). These objectives were especially relevant in pancreatic cancer, glioblastoma, ovarian cancer, metastatic breast cancer, and other difficult-to-treat tumors, where treatment failure is linked not only to inadequate drug potency but also to restricted delivery and adaptive biological resistance. Thus, barrier-overcoming strategies connect stimuli-responsive engineering to the practical therapeutic requirement of reaching, sensitizing, and treating tumor regions that are poorly addressed by passive accumulation alone (Samson et al., 2020; Wang L. J. et al., 2025; Wang et al., 2024b; Zhang F. et al., 2023; Zhang et al., 2022; Puzzo et al., 2025; Puzzo et al., 2024).

### Therapeutic index, safety, and localized precision

Improving the therapeutic index was a cross-cutting objective throughout the reviewed studies. Responsive platforms were designed to reduce systemic toxicity, minimize premature release, limit off-target exposure, protect normal tissues, and, where applicable, support localized or site-specific therapy (Gupta et al., 2024; Jia et al., 2020; Li et al., 2024; Miao et al., 2020; Perera et al., 2022; Raj and Sundaramoorthy, 2025; Vaghasiya et al., 2021; Wang L. et al., 2025; Xiao et al., 2021; Yang et al., 2020; Zhang D. et al., 2024; Zhang Y. P. et al., 2025). This safety-oriented objective is integral to precision cancer therapy because therapeutic precision depends on selective benefit as well as reduced harm outside the intended site of action.

Several studies further addressed treatment-limiting safety concerns by emphasizing controlled activation, reduced systemic side effects, safer clearance, localized administration, or improved therapeutic accumulation in cervical, lung, liver, breast, colorectal, and other solid tumor therapies (Kv et al., 2020; Pandya et al., 2025; Pragti et al., 2023; Rashidzadeh et al., 2023; Tao and Yin, 2020; Wei et al., 2024; Ye et al., 2020). These objectives indicate that stimuli-responsive bioengineering is being used not only to intensify antitumor activity but also to refine the balance between efficacy and tolerability in therapies constrained by toxicity, rapid clearance, premature leakage, poor tissue specificity, or long-term retention.

Taken together, the reviewed studies define a coherent therapeutic bioengineering objective: to create cancer-directed platforms that respond to disease-relevant biological cues, improve controlled and intracellular therapeutic action, coordinate multimodal treatment, overcome tumor barriers and resistance, and enhance antitumor efficacy while reducing off-target toxicity. Within the framework of stimuli-responsive bioengineered platforms for precision cancer therapy, the most important contribution of these systems is their ability to couple therapeutic action to the biological conditions of malignant tissues, thereby improving the selectivity, accessibility, and functional precision of cancer treatment.

## Bioengineered platform architecture

**FIGURE 2:**
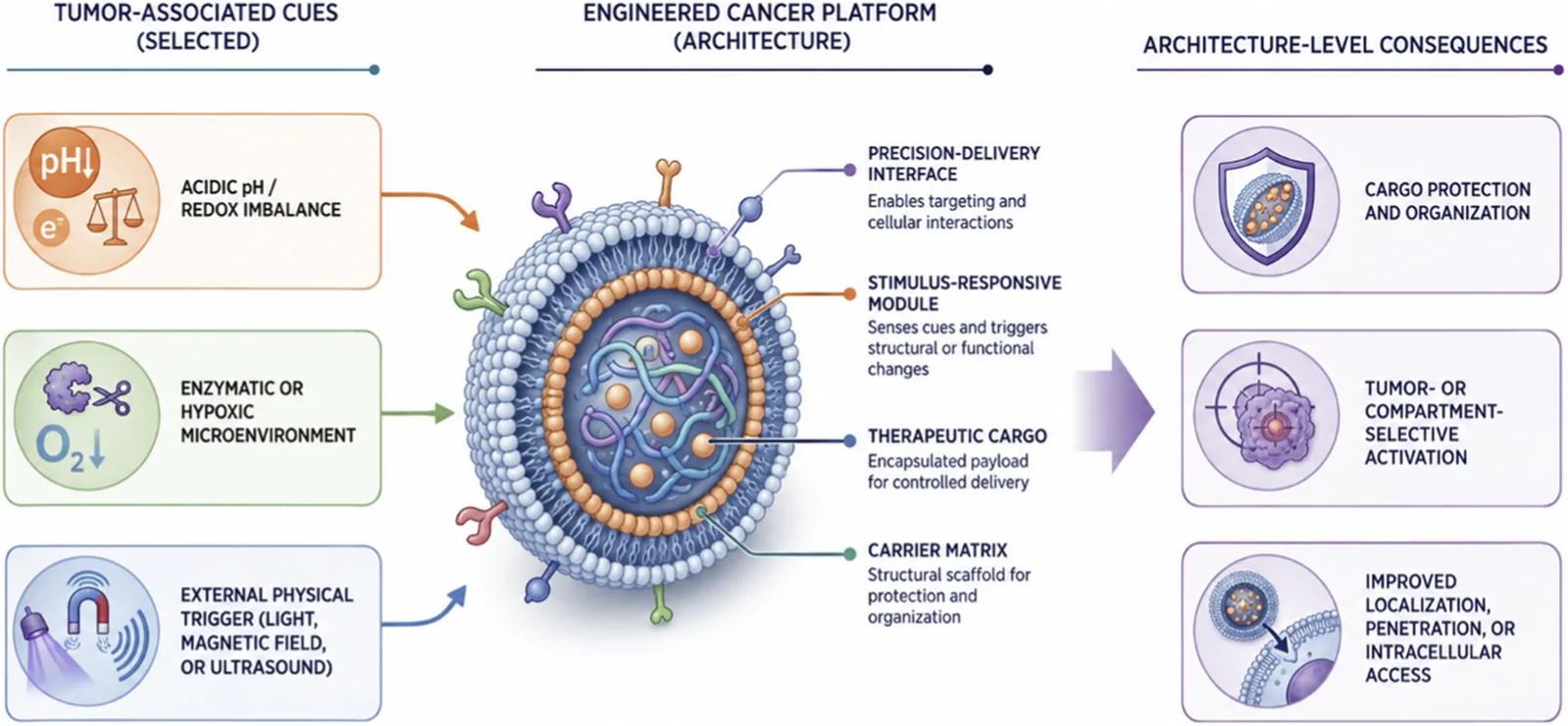
Modular Architecture of Stimuli-Responsive Bioengineered Platforms. This schematic demonstrates how stimuli-responsive precision is physically encoded into a bioengineered delivery platform. The carrier matrix forms the structural body, while therapeutic or theranostic cargo is organized within the core reservoir. Stimulus-responsive modules, including functional linkers, gates, coatings, and cleavable layers, regulate access in response to biochemical or external cues. Surface ligands, shells, charge-switching groups, and targeting motifs create the precision-delivery interface. Together, these modular components define responsive access, targeted interfacing, and organized cargo presentation at the architectural level (Prepared with the assistance of Gemini’s NotebookLM Pro and ChatGPT Plus).

Stimuli-responsive bioengineered platforms for precision cancer therapy are best understood as integrated delivery architectures rather than passive drug containers. In this section, the carrier matrix denotes the structural material that organizes the system; the cargo denotes the therapeutic or theranostic payload; the stimulus-responsive module denotes the component that responds to tumor-associated or externally applied cues; and the precision-delivery interface denotes the ligand, coating, shell, charge-switching element, or transport feature that supports selective localization, penetration, or intracellular access. Across the reviewed systems, these components were configured to respond to pH, redox potential, glutathione, enzymes, hypoxia, reactive oxygen species, temperature, magnetic fields, light, ultrasound, or charge transitions. This architecture-to-precision logic is summarized in Figure 2. The platform diversity was substantial, spanning biomacromolecular nanoparticles, hydrogels, nanogels, polymeric micelles, vesicles, prodrug nanoparticles, lipid-based systems, mesoporous silica, organosilica, metal–organic frameworks, covalent organic frameworks, magnetic nanocomposites, carbon-based materials, and other hybrid inorganic–organic constructs. Despite this diversity, the central design logic was consistent: the scaffold organized the payload, while responsive linkers, coatings, ligands, shells, gates, or physical components encoded the platform’s precision-delivery function.

### Biomacromolecular and protein-derived platforms

Natural and semi-natural biomacromolecules provided an important architectural basis for stimuli-responsive delivery because they could function simultaneously as structural scaffolds, biological interfaces, and carriers for chemical or nucleic-acid payloads. Albumin, sericin, dextran, keratin, hyaluronic acid, gelatin, chitosan, zein, lysozyme–hyaluronan composites, plant-derived proteins, and protein cages were used to support responsive or targeted drug delivery. Bovine serum albumin/zeolitic imidazolate framework-8 hybrid nanocarriers combined bovine serum albumin with a zeolitic imidazolate framework and carried gemcitabine and amygdalin while incorporating polydopamine, Au^3+^, and gallic-acid functional modules (Abd Al-Jabbar et al., 2022). Sericin-based nanocarriers were designed around charge-reversal behavior for resveratrol and melatonin delivery (Aghaz et al., 2023), whereas dextran vesicular prodrug systems carried doxorubicin and camptothecin (Curcio et al., 2020), and keratin–polyethylene glycol micelles carried doxorubicin within a protein-derived micellar architecture (Du et al., 2020).

Hyaluronic acid-containing systems were especially prominent because hyaluronic acid could serve as both a biomaterial component and a targeting-associated interface. Hyaluronic acid-based prodrug micelles incorporated celastrol, glycyrrhetinic acid, dasatinib/cholesterol components, or doxorubicin, linking biomacromolecular structure to redox-responsive or receptor-associated design (Fu et al., 2025; Lin et al., 2021). Other representative biomacromolecular platforms included lysozyme–hyaluronan colloidal nanoparticles carrying 5-fluorouracil and curcumin, with a reported size of 126.1 nm and polydispersity index of 0.1 (Li et al., 2024); glucose-functionalized polydopamine nanoparticles carrying bortezomib with glucosyl ligands for glucose transporter 1–oriented targeting (Li Y. P. et al., 2020); *Camellia oleifera* protein nanoparticles associated with doxorubicin delivery (Qian et al., 2020); multilayered small heat-shock-protein cage nanoparticles carrying paclitaxel with peptide and coating components (Shi et al., 2020); dopamine-modified hyaluronic acid/copper (II)/6-mercaptopurine coordination polymer prodrugs with a particle size of 173.5 nm, drug loading of 49.5 mg/g, and encapsulation efficiency of 70.18% (Tao and Yin, 2020); gelatin nanoparticles decorated with concanavalin A for cisplatin delivery (Vaghasiya et al., 2021); and folic-acid-modified, bovine serum albumin-cloaked hollow mesoporous silica nanoparticles carrying indocyanine green and paclitaxel (Zhang and Liang, 2024). Together, these platforms show how biological matrices can contribute not only to carrier construction but also to ligand display, charge adaptation, coordination chemistry, and stimulus-responsive cargo organization.

### Hydrogel, nanogel, and injectable matrix platforms

Hydrogel and nanogel systems used hydrated or crosslinked networks to integrate therapeutic payloads with local responsiveness. Their architectural role differed from compact nanoparticles because the polymeric network itself often served as the cargo-retaining and stimulus-responsive structure. Chitosan hydrogels containing mesoporous silica nanoparticles were used for co-delivery of 5-fluorouracil and everolimus (Arvejeh et al., 2025), while chitosan/gelatin/gold nanoparticle hybrid nanogels carried doxorubicin, with the final chitosan/gold nanoparticle@gelatin system reported at 119.3 nm and doxorubicin loading of approximately 56% (Aslzad et al., 2023). Related networked systems included hyaluronic acid/carboxymethyl dextran/gelatin methacryloyl nanogels carrying bosutinib (Bhattacharya et al., 2024), alpha-cyclodextrin/polyethylene glycol-micelle supramolecular hydrogels carrying 8-hydroxyquinoline glycoconjugates and doxorubicin (Dominski et al., 2022), and fluorescein O,O′-diacrylate-crosslinked anionic and cationic fluorescent nanogels carrying cisplatin and 5-fluorouracil (González-Urías et al., 2021).

More complex matrix-based constructs integrated inorganic shells, imaging elements, proteins, or gene-silencing cargo into the same responsive architecture. Hybrid nanoparticles combined crosslinked methoxy poly (ethylene glycol)-g-poly (aspartic acid)-g-tyrosine, a disulfide-crosslinked interlayer, a calcium phosphate shell, curcumin, and doxorubicin hydrochloride (Li et al., 2021). Nanocomposite hydrogels incorporated polyethyleneimine-modified mesoporous silica nanoparticles, cisplatin, short hairpin RNA targeting protein arginine methyltransferase 5, and methacryloylated hyaluronic acid (Liu et al., 2025). Iron oxide nanoflower/chitosan nanogels carried doxorubicin with a loading capacity of 67.3% ± 5.7% and efficiency of 84.1% ± 7.2% (Ozkahraman et al., 2026), while ionic-liquid-containing hydrogels incorporated [TMG][Ol], 5-fluorouracil, and heparin (Pandya et al., 2025). Other matrix architectures included amine-functionalized cellulose nanogels carrying doxorubicin with 86.7% loading efficiency (Raj and Sundaramoorthy, 2025), poly (methacrylic acid)-based nanohydrogels carrying methotrexate and chloroquine with particle sizes below 50 nm and loading capacities of 4.99% and 25.01%, respectively (Rashidzadeh et al., 2023), acid-responsive bromelain/doxorubicin nanogels (Wang L. J. et al., 2025), chlorin e6-functionalized dynamically crosslinked nanogels carrying cytochrome c and chlorin e6 (Yang et al., 2022), and generation 3 poly (amidoamine) dendrimer nanogels containing gold nanoparticles and toyocamycin with a reported size of 193 nm (Zhang G. Z. et al., 2023). These examples illustrate the specific contribution of hydrogel and nanogel architectures: they provide spatially organized networks in which drug molecules, proteins, enzymes, nucleic acids, photosensitizers, or imaging components can be retained within one responsive construct.

### Polymeric micelles, vesicles, and prodrug nanoparticles

Polymeric micelles, vesicles, and prodrug nanoparticles formed the most modular group of stimuli-responsive architectures. Their design typically relied on amphiphilic block copolymers, prodrug backbones, cleavable linkers, charge-switchable segments, ligand-bearing shells, or responsive subsurface layers. This modularity allowed the scaffold, cargo, targeting element, and stimulus-response component to be combined within the same nanoscale construct. Representative systems included poly (N-isopropylacrylamide)-disulfide-block-poly (lysine)-block-poly (ε-caprolactone) triblock copolymer micelles carrying doxorubicin, with a micellar size of approximately 200 nm (Augustine et al., 2020); sulfobetaine methacrylate-functionalized poly (lactic acid)/PEAd nanoparticles carrying paclitaxel, with polymer ratios of 95/5, 90/10, and 75/25 w/w and particle sizes of 220–565 nm (Christodoulou et al., 2025); nanocomplexes carrying SN-38 and celecoxib (Gao et al., 2025); core-crosslinked methoxy poly (ethylene glycol)-poly [aspartic acid-(hydrazone-doxorubicin)] micelles carrying SN-38, with drug loading of 7.32% and encapsulation efficiency of 86.23% (Gebrie et al., 2022); and charge-switchable nanoparticles combining a polyethylene glycol–poly-L-lysine–doxorubicin conjugate with lapatinib (Guo et al., 2020).

Within this same family, prodrug and co-delivery systems were used to integrate chemotherapy, gene modulation, immune modulation, or externally activated therapy into the platform architecture. Examples included folic-acid-targeted nanoparticles co-delivering pirarubicin and salinomycin (Gupta et al., 2024), programmed PEOz-b-PSD/poly (amidoamine)/doxorubicin nanoparticles with pKa 6.96 (Jia et al., 2020), paclitaxel/doxorubicin polyprodrug micelles with paclitaxel loading of 25.6% and critical micelle concentration of approximately 3.16 mg/L (Jiang et al., 2020), estradiol-conjugated polymersomes carrying doxorubicin (Mamnoon et al., 2020), poly (acrylic acid)-block-polyethylene glycol-block-poly (acrylic acid)/MnO_2_ vesicles with an approximately 30 nm diameter and doxorubicin loading capacity near 94% (Miao et al., 2020), beta-cyclodextrin-grafted poly (maleate-co-poly [lactic-co-glycolic acid]) micelles carrying doxorubicin and conferone with near-100% encapsulation efficiency (Rahmani et al., 2021), triblock copolymer micelles carrying paclitaxel and small interfering RNA (Shi et al., 2021), enzyme/reduction dual-responsive folate micelles carrying doxorubicin (Wan et al., 2024), luteinizing hormone-releasing hormone-targeted disulfide-crosslinked micelles carrying paclitaxel (Xiao et al., 2021), chitosan-based vectors carrying doxorubicin and Bcl-2 small interfering RNA (Yan et al., 2020), hyaluronic acid/poly (beta-amino ester) nanoprodrugs carrying gemcitabine and microRNA-21 small interfering RNA (Zhang F. et al., 2023), angiopep-2-modified micelles carrying Dbait and doxorubicin (Zhang et al., 2022), ultrasound-responsive conjugated polymer nanoparticles carrying camptothecin (Zhang Y. P. et al., 2025), and COA-modified four-arm polyethylene glycol nanomicelles carrying paclitaxel (Zhou et al., 2023). These platforms demonstrate how polymer design can encode responsiveness through disulfide linkers, pH-sensitive blocks, reactive oxygen species-responsive units, enzyme-cleavable peptides, and ligand-modified surfaces while accommodating small molecules, prodrugs, small interfering RNA, microRNA, radiosensitizers, or immune-related cargos.

### Inorganic, magnetic, silica, MOF, carbon-based, and hybrid composite platforms

Inorganic and hybrid platforms added architectural functions that are less readily achieved with purely organic carriers, including mesoporosity, magnetic responsiveness, metal-mediated functionality, photothermal or photodynamic activity, pore gating, and imaging integration. Magnetic and metal-containing systems included Fe_3_O_4_@OA-CS-5-FLU nanoparticles carrying 5-fluorouracil (Ayyanaar et al., 2020), mesoporous magnetic graphene quantum dot systems carrying paclitaxel (Bilchi et al., 2025), copper-based nanoplatforms combining a poly (amino acid) disulfiram prodrug framework with CuO_2_ nanoparticles (Cheng et al., 2023), disulfide-containing magnetic nanocarriers incorporating Fe_3_O_4_, SiO_2_, oleic acid, Pluronic F127, L-cysteine, carboxymethyl-beta-cyclodextrin, folic acid, and doxorubicin (Ehsanimehr et al., 2025), and CuFe_2_O_4_@poly (methacrylic acid)@Lig-ADH nanocarriers carrying curcumin with an average diameter of approximately 15 nm (Fattahi et al., 2024). Other inorganic hybrid constructs included TiO_2_@poly (acrylic acid)-calcium phosphate nanoparticles carrying doxorubicin (Han et al., 2022), branched polyethyleneimine-disulfide-platinum/hyaluronidase@calcium phosphate nanocomplexes containing cisplatin, hyaluronidase, and amorphous calcium phosphate with a size of 143 ± 14 nm (Jia et al., 2022), perfluorooctyl bromide oil-droplet nanoparticles carrying oxygen, IR780, and mTHPC (Kv et al., 2020), Fe_3_O_4_@polyethyleneimine-curcumin nanoparticles with a size of 20–30 nm and encapsulation efficiency of 89.10% under acidic conditions (Sasikala et al., 2025), disulfide-linked polyethylene glycol-polycaprolactone@iron-doxorubicin nanodrugs combining a disulfide-linked amphiphilic polymer with an iron-doxorubicin complex (Wang et al., 2026), charge-reversal nanoplatforms carrying mitoxantrone and Cu^2+^ with cyclic cRGDfK and r9 peptide components (Tan et al., 2024), and glutathione-responsive magnetic mesoporous silica nanoparticles incorporating disulfide organosilicon grafting and doxorubicin (Zhang D. et al., 2024).

Mesoporous silica, organosilica, metal–organic framework, covalent organic framework, and carbon-based architectures were frequently used when the platform required cargo accommodation together with gated retention or additional physical functionality. Doxorubicin-loaded mesoporous silica nanoparticles incorporated polyethyleneimine and anisamide (Chang et al., 2022). Copper sulfide/metformin mesoporous silica systems combined polydopamine and copper sulfide photothermal elements (Chen et al., 2026), while carboxyl-functionalized mesoporous silica/hyperbranched polymer platforms incorporated bicalutamide and folic acid (Feng et al., 2023). Lignin@graphene oxide@zeolitic imidazolate framework-8 nanocarriers combined lignin, graphene oxide, zeolitic imidazolate framework-8, 5-fluorouracil, and metformin (Gorish et al., 2026), and polymer-coated metal–organic framework nanoparticles carried doxorubicin and cisplatin (Hu et al., 2022). Other representative systems included pollen-like hollow silica nanoparticles carrying doxorubicin and an aptamer, with a void diameter of 292 ± 14 nm, pore size of 11.8 nm, and drug-loading efficiency of 0.509 g/g (Jin R. R. et al., 2021); Zn-MOF-74 and MOF-808 systems carrying 5-fluorouracil with sodium alginate or polydopamine coatings (Kazemi et al., 2025a; Kazemi et al., 2025b); phenylboronic-acid-functionalized, gold-gated mesoporous silica nanoparticles carrying compound 1j, with a mean diameter of approximately 86 nm and drug-loading content of 13.68% (Kundu et al., 2021); SBA-15 mesoporous silica nanoparticles incorporating enzalutamide, folic acid, thiolated nitrogen-doped carbon dots, disulfide linkages, and drug-loading efficiency of 69.95% (Mahani et al., 2025); iron-based carbon-dot metal–organic gels carrying 5-fluorouracil with loading capacity of 72.9% (Maiti et al., 2025); UiO-66(Zr)-NH_2_ and UiO-66(Zr)-His nanoparticles carrying 5-fluorouracil (Migasova et al., 2025); porous silica nanoparticles with sizes of 42–178 nm, pore structures of 1.1–2.7 nm, and doxorubicin loading of 22% w/w (Newham et al., 2021); rice-husk-derived mesoporous silica nanoparticles functionalized with acrylic acid and N-isopropyl acrylamide carrying SN-38 (Nie et al., 2023); copper-ligand mesoporous silica nanoparticles carrying curcumin and incorporating a heterocyclic Schiff-base copper (II) complex (Sujatha et al., 2025); azobenzene-containing covalent organic framework nanocarriers carrying doxorubicin and modified with a sulfamide-based zwitterionic polymer (Wang et al., 2024a); galactosamine/polydopamine/mesoporous silica platforms carrying aspirin (Wei et al., 2024); lipid-coated CaCO_3_ nanoparticles carrying doxorubicin and oxaliplatin prodrugs (Xu et al., 2022); biodegradable hollow mesoporous organosilica nanoparticles carrying doxorubicin (Yang et al., 2020); mesoporous silica/polyacrylic acid/pH-sensitive lipid/F56 peptide systems carrying paclitaxel and arsenic trioxide (Zhang B. B. et al., 2021); and CaCO_3_ nanoparticles incorporating mitoxantrone and celastrol (Zhang L. et al., 2024). In this group, the architecture itself often supplied the functional reservoir, gate, imaging element, magnetic component, photothermal module, or metal-associated therapeutic interface.

### Liposomal, polymer–lipid, oil-droplet, and membrane-like systems

Membrane-like platforms bridged conventional nanocarrier design and stimuli-responsive precision delivery by combining lipid or lipid-like stability with tumor-responsive cargo access. Layered smart nanoparticles co-delivering paclitaxel and vascular endothelial growth factor small interfering RNA had a reported size of 85.25 nm, polydispersity index of 0.261, and zeta potential of 5.25 mV (Jin M. J. et al., 2021). Perfluorooctyl bromide oil-droplet nanoparticles consisted of lipid membrane-enclosed perfluorooctyl bromide droplets modified with N-acetyl histidine-modified D-α-tocopheryl polyethylene glycol 1,000 succinate and carried oxygen, IR780, and mTHPC (Kv et al., 2020). Stealth nanoparticles carried paclitaxel and an anti–programmed cell death protein 1 antibody with a choline-based ionic-liquid coating (Li et al., 2025), while polymer–lipid hybrid nanoparticles were composed of poly (lactic-co-glycolic acid), 1,2-distearoyl-sn-glycero-3-phosphoethanolamine-methoxy polyethylene glycol, and cyclic arginine–glycine–aspartic acid-modified polyethylene glycol lipid for co-delivery of LTX-315 and transforming growth factor beta 1 small interfering RNA (Phung et al., 2023). Tumor-activated pH-sensitive liposomes carried a hypoxia-cleavable Exatecan-squalene prodrug and IR808 with a uniform size of approximately 136 nm (Wang L. et al., 2025). Lipid-coated CaCO_3_ nanoparticles combined doxorubicin, amphiphilic oxaliplatin prodrugs, commercial lipids, and polyethylene glycol (Xu et al., 2022). These architectures show how lipid shells, membrane-like coatings, pH-sensitive lipids, ionic-liquid layers, hypoxia-cleavable prodrugs, oxygen-carrying droplets, and peptide-modified lipid surfaces can be integrated into responsive systems for chemo-, immune-, photodynamic-, or oxygen-associated delivery.

### Barrier-focused and microenvironment-adaptive delivery architectures

A smaller group of references addressed platform architecture at the level of tumor-barrier navigation rather than as a single material formulation. Hypoxia-responsive nanoparticles were described as systems built around bioreductive molecular structures and hypoxia-associated enzymatic responsiveness (Li Y. C. et al., 2020). Size-defined liposomal nanoparticle models measuring approximately 126, 164, and 234 nm were used as architectural models for spatial distribution in tumor tissue (Samson et al., 2020). Nanoparticle-mediated tumor-microenvironment remodeling platforms were described as systems designed to target endothelial cells, immune cells, cancer-associated fibroblasts, and extracellular matrix components (Tang et al., 2021). Cell-mediated delivery was represented by nanoparticle-loaded macrophages evaluated within a three-dimensional tumor-microenvironment-on-a-chip gel matrix (Wang et al., 2020). Stimuli-responsive transcytosis-based nanocarriers were discussed as an architectural strategy for tumor penetration without specification of a single material composition or payload (Wang et al., 2024b). These examples broaden the definition of platform architecture by showing that precision delivery can be shaped not only by particle composition but also by how the carrier interfaces with vascular distance, hypoxic regions, stromal barriers, immune compartments, and intracellular transport pathways.

Overall, the reviewed platforms demonstrate that stimuli-responsive bioengineering is fundamentally an architectural strategy. Precision is encoded through the coordinated arrangement of scaffold, cargo, stimulus-responsive element, and targeting or navigation module. This framing helps distinguish platform architecture from downstream biological performance: the architecture defines how therapeutic payloads are organized, protected, directed, and made responsive to tumor-associated environments, whereas efficacy, mechanism, and translational performance require separate evaluation in the corresponding sections. Table 2 summarizes these architecture-level patterns across the major platform classes.

**TABLE 2:** Stimuli-responsive architectural patterns in bioengineered platforms for precision cancer therapy.

Bioengineered platform architecture	Stimulus-responsive architectural module	Precision-therapy function encoded in the design	Architecture-linked technical/formulation data retained in this section	Representative references
Biomacromolecular and protein-derived nanoparticles	Biopolymer shells or matrices incorporating pH-responsive, redox-responsive, charge-reversal, coordination, or receptor-facing elements	Uses biological or semi-biological scaffolds as both carrier structures and precision-delivery interfaces, especially where hyaluronic acid, glucose, folate, gelatin, albumin, or protein cages contribute to targeting or responsiveness	Lysozyme–hyaluronan nanoparticles: 126.1 nm, polydispersity index of 0.1; dopamine-modified hyaluronic acid/copper (II)/6-mercaptopurine coordination polymer prodrugs: 173.5 nm, 49.5 mg/g drug loading, and 70.18% encapsulation efficiency; gelatin–concanavalin A nanoparticles; bovine serum albumin-cloaked hollow mesoporous silica nanoparticles with folic acid	(Abd Al-Jabbar et al., 2022; Aghaz et al., 2023; Curcio et al., 2020; Du et al., 2020; Fu et al., 2025; Li et al., 2024; Li et al., 2020b; Lin et al., 2021; Qian et al., 2020; Shi et al., 2020; Tao and Yin, 2020; Vaghasiya et al., 2021; Zhang and Liang, 2024)
Hydrogel, nanogel, and injectable matrix platforms	Crosslinked or supramolecular networks containing pH-sensitive, redox-cleavable, enzyme-responsive, ionic-liquid, photodynamic, or imaging-associated components	Provides matrix-based cargo retention and localized multi-cargo integration, making these systems suitable for drugs, proteins, enzymes, photosensitizers, and nucleic acids within one responsive construct	Chitosan/gold nanoparticle@gelatin system: 119.3 nm and approximately 56% doxorubicin loading; iron oxide nanoflower/chitosan nanogels: 67.3% ± 5.7% loading capacity and 84.1% ± 7.2% efficiency; cellulose nanogel: 86.7% doxorubicin loading; poly (methacrylic acid) nanohydrogels: below 50 nm, 4.99% methotrexate loading, and 25.01% chloroquine loading	(Arvejeh et al., 2025; Aslzad et al., 2023; Bhattacharya et al., 2024; Dominski et al., 2022; González-Urías et al., 2021; Li et al., 2021; Liu et al., 2025; Ozkahraman et al., 2026; Pandya et al., 2025; Raj and Sundaramoorthy, 2025; Rashidzadeh et al., 2023; Wang et al., 2025b; Yang et al., 2022; Zhang et al., 2023b)
Polymeric micelles, vesicles, and prodrug nanoparticles	Amphiphilic blocks, disulfide linkers, pH-sensitive segments, charge-switchable groups, reactive oxygen species-responsive units, enzyme-cleavable peptides, or ligand-modified shells	Offers modular architecture for combining scaffold design, multi-drug loading, nucleic-acid delivery, receptor targeting, and intracellular stimulus response	Triblock micelles: approximately 200 nm; poly (lactic acid)/PEAd-sulfobetaine methacrylate nanoparticles: 220–565 nm; core-crosslinked micelles: 7.32% SN-38 loading and 86.23% encapsulation efficiency; poly (acrylic acid)-block-polyethylene glycol-block-poly (acrylic acid)/MnO_2_ vesicles: approximately 30 nm and approximately 94% doxorubicin loading; paclitaxel/doxorubicin polyprodrug micelles: 25.6% paclitaxel loading and critical micelle concentration of approximately 3.16 mg/L	(Augustine et al., 2020; Christodoulou et al., 2025; Gao et al., 2025; Gebrie et al., 2022; Guo et al., 2020; Gupta et al., 2024; Jia et al., 2020; Jiang et al., 2020; Mamnoon et al., 2020; Miao et al., 2020; Rahmani et al., 2021; Shi et al., 2021; Wan et al., 2024; Xiao et al., 2021; Yan et al., 2020; Zhang et al., 2023a; Zhang et al., 2022; Zhang et al., 2025b; Zhou et al., 2023)
Inorganic, magnetic, and metal-containing hybrid composites	Magnetic cores, metal oxide components, redox-sensitive metal coordination, photothermal or photodynamic agents, charge-reversal groups, or peptide-functionalized surfaces	Adds physical or chemical functions beyond cargo carriage, including magnetic guidance, metal-associated functionality, imaging, reactive oxygen species-related functionality, or deep-penetration design	CuFe_2_O_4_@poly (methacrylic acid)@Lig-ADH nanocarriers: approximately 15 nm; branched polyethyleneimine-disulfide-platinum/hyaluronidase@calcium phosphate nanocomplexes: 143 ± 14 nm; Fe_3_O_4_@polyethyleneimine-curcumin nanoparticles: 20–30 nm and 89.10% encapsulation efficiency; charge-reversal nanoplatforms included mitoxantrone, Cu^2+^, cyclic cRGDfK, and r9 peptides	(Ayyanaar et al., 2020; Bilchi et al., 2025; Cheng et al., 2023; Ehsanimehr et al., 2025; Fattahi et al., 2024; Han et al., 2022; Jia et al., 2022; Kv et al., 2020; Sasikala et al., 2025; Tan et al., 2024; Wang et al., 2026; Zhang et al., 2024a)
Mesoporous silica, organosilica, metal–organic framework, covalent organic framework, and carbon-based platforms	Mesoporous reservoirs, pH-sensitive coatings, redox-responsive gates, polydopamine layers, metal–organic coordination, azobenzene groups, carbon dots, or cleavable organosilica frameworks	Prioritizes cargo accommodation, gated retention, imaging integration, and hybrid chemo-photothermal or chemo-photodynamic platform design	Pollen-like hollow silica: 292 ± 14 nm void diameter, 11.8 nm pores, and 0.509 g/g loading; phenylboronic-acid/gold-gated mesoporous silica nanoparticles: approximately 86 nm and 13.68% loading; SBA-15 mesoporous silica nanoparticles: 69.95% drug loading; iron-based carbon-dot metal–organic gel: 72.9% loading; porous silica: 42–178 nm, 1.1–2.7 nm pores, and 22% w/w doxorubicin loading	(Chang et al., 2022; Chen et al., 2026; Feng et al., 2023; Gorish et al., 2026; Hu et al., 2022; Jin et al., 2021b; Kazemi et al., 2025a; Kazemi et al., 2025b; Kundu et al., 2021; Mahani et al., 2025; Maiti et al., 2025; Migasova et al., 2025; Newham et al., 2021; Nie et al., 2023; Sujatha et al., 2025; Wang et al., 2024a; Wei et al., 2024; Yang et al., 2020; Zhang et al., 2021a)
Liposomal, polymer–lipid, oil-droplet, and membrane-like systems	pH-sensitive lipids, lipid shells, ionic-liquid coatings, hypoxia-cleavable prodrugs, oxygen-carrying droplets, antibody or small interfering RNA cargo modules, and peptide-modified lipid surfaces	Bridges conventional nanocarrier stability with tumor-triggered access to chemotherapy, immunotherapy, photodynamic therapy, or oxygen-associated payloads	Layered paclitaxel/vascular endothelial growth factor small interfering RNA nanoparticles: 85.25 nm, polydispersity index of 0.261, and zeta potential of 5.25 mV; perfluorooctyl bromide oil-droplet nanoparticles carried oxygen, IR780, and meta-tetra (hydroxyphenyl)chlorin; pH-sensitive Exatecan/IR808 liposomes: approximately 136 nm; lipid-coated CaCO_3_ platform combined doxorubicin and a platinum (IV) prodrug	(Jin et al., 2021a; Kv et al., 2020; Li et al., 2025; Phung et al., 2023; Wang et al., 2025a; Xu et al., 2022)
Barrier-focused and microenvironment-adaptive delivery architectures	Hypoxia-responsive structures, size-defined liposomal models, cell-mediated transport, stromal or tumor-microenvironment targeting, and transcytosis-oriented design	Treats tumor penetration, vascular distance, hypoxic niches, immune compartments, or stromal barriers as part of the platform architecture rather than as downstream outcomes alone	Liposomal models: approximately 126, 164, and 234 nm; nanoparticle-loaded macrophages evaluated in a three-dimensional tumor-microenvironment-on-a-chip gel matrix; review-level platforms targeted endothelial cells, immune cells, cancer-associated fibroblasts, extracellular matrix, or transcytosis pathways	(Li et al., 2020a; Samson et al., 2020; Tang et al., 2021; Wang et al., 2020; Wang et al., 2024b)

## Engineered functional mechanism

**FIGURE 3:**
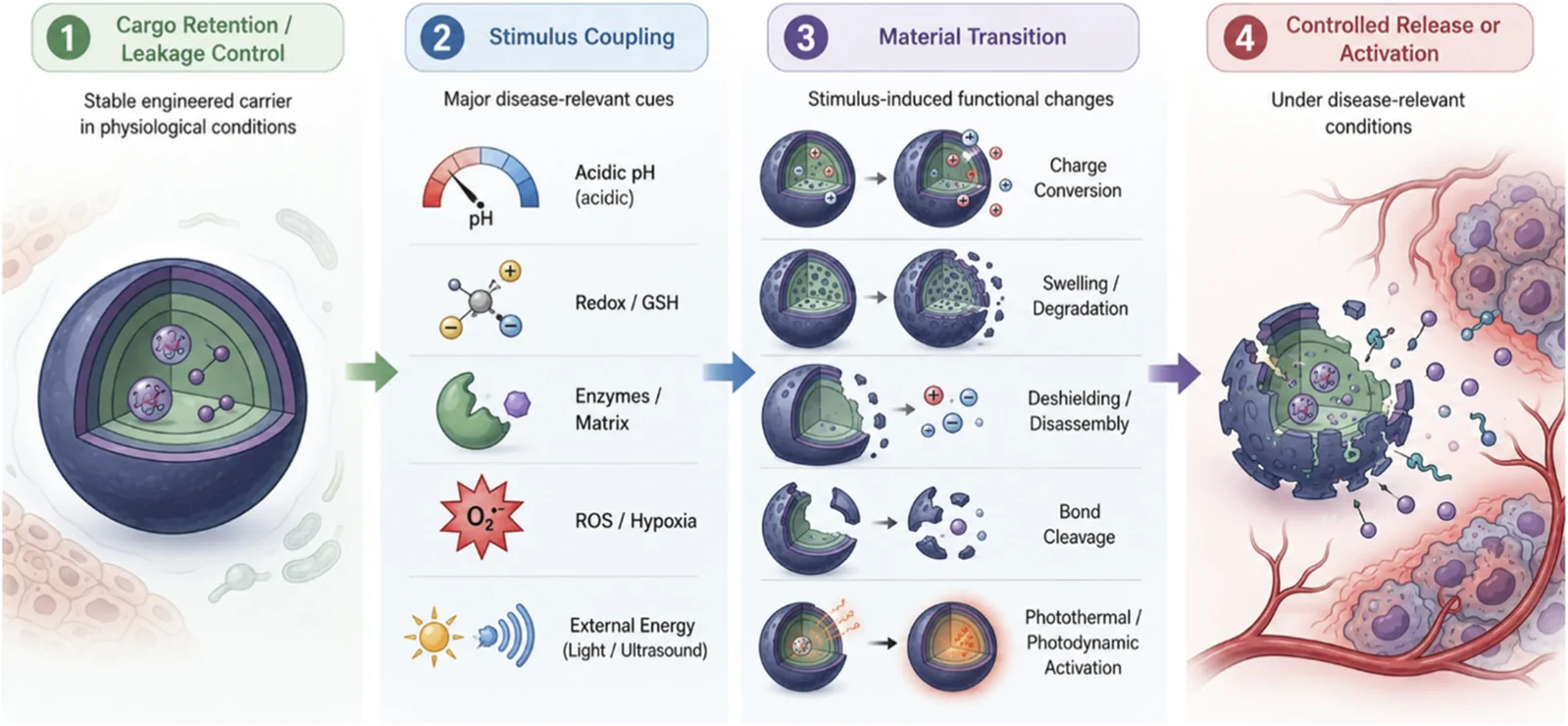
Stimulus–Material Coupling Governing Engineered Functional Mechanisms. This figure demonstrates how engineered carriers maintain payload retention under physiological conditions to minimize premature leakage. Disease-relevant stimuli, including acidic pH, redox imbalance, glutathione, enzymes, matrix cues, reactive oxygen species, hypoxia, light, and ultrasound, couple with responsive material features. These cues trigger material transitions such as swelling, degradation, charge conversion, deshielding, disassembly, bond cleavage, and photodynamic or photothermal activation. As a result, the carrier becomes release-active, enabling controlled payload release and activation within the local microenvironment. The schematic emphasizes the causal mechanism linking stimulus recognition, material transformation, and controlled functional release (Prepared with the assistance of Gemini’s NotebookLM Pro and ChatGPT Plus).

Stimuli-responsive bioengineered platforms for precision cancer therapy are designed to convert tumor-associated or externally applied cues into controlled material responses. In this context, a stimulus-responsive mechanism refers to a programmed change in the carrier, such as release, swelling, degradation, charge conversion, deshielding, disassembly, photothermal conversion, photodynamic activation, or enzyme-mediated remodeling, that occurs after exposure to a defined biological or physical trigger. Across the reviewed studies, precision was not based on passive encapsulation alone; rather, it depended on whether a carrier could preserve its cargo under physiological exposure and then become release-active under disease-relevant conditions. These mechanisms were implemented across micelles, hydrogels, nanogels, metal–organic frameworks, mesoporous silica, liposomes, protein-based particles, polymeric vesicles, prodrug assemblies, and hybrid inorganic–organic nanocarriers (Abd Al-Jabbar et al., 2022; Augustine et al., 2020; Chen et al., 2026; Gupta et al., 2022; Jin R. R. et al., 2021; Mamnoon et al., 2020; Ozkahraman et al., 2026; Tao and Yin, 2020; Xiao et al., 2021; Zhang Y. P. et al., 2025). The central engineering logic is stimulus–material coupling: each platform uses a biological or externally controlled input to regulate when, where, and how the delivery system changes its functional state. This sequence from cargo retention to stimulus-triggered transition and controlled release is illustrated in Figure 3.

### Acidic pH as a trigger for release, degradation, and charge conversion

Acid-responsive delivery was the most frequently used mechanism and provides the clearest example of stimulus-matched carrier design. Acidic tumor, endosomal, or lysosomal environments were used to trigger protonation, matrix swelling, framework dissolution, shell degradation, hydrogel destabilization, micellar dissociation, or accelerated release. Representative systems included zeolitic imidazolate framework-8-coated albumin carriers, sericin-based nanoparticles, hyaluronic acid/carboxymethyl dextran/gelatin methacryloyl nanogels, pH-sensitive mesoporous silica and metal–organic framework systems, CaCO_3_-based particles, polymeric micelles, cellulose nanogels, and injectable hydrogels (Abd Al-Jabbar et al., 2022; Aghaz et al., 2023; Bhattacharya et al., 2024; Hu et al., 2022; Jin R. R. et al., 2021; Kazemi et al., 2025b; Ozkahraman et al., 2026; Pandya et al., 2025; Raj and Sundaramoorthy, 2025; Wei et al., 2024; Xu et al., 2022). The functional value of these platforms lies in their ability to restrain premature release under physiological conditions while increasing payload liberation under acidic disease-relevant conditions.

Several studies provided quantitative support for this mechanism. Sericin nanocarriers released more drug at pH 6.0 than at pH 6.5 or physiological pH, while also shifting from a negative surface charge under physiological conditions toward a positive charge in acidic media (Aghaz et al., 2023). Hyaluronic acid/carboxymethyl dextran/gelatin methacryloyl nanogels achieved 80%–90% entrapment, particle sizes of 100–200 nm, and sustained release at pH 5.0 (Bhattacharya et al., 2024). Pollen-like hollow silica particles combined a 292 ± 14 nm internal void, 11.8 nm surface pores, 0.509 g/g loading, and release up to 87.5% at pH 5.0 (Jin R. R. et al., 2021). MOF-808%–15% released 70.8% at pH 5.5 compared with 57.7% at pH 7.4, with drug–framework interaction supported by an adsorption energy of −1.13 eV (Kazemi et al., 2025b). Ionic-liquid hydrogels released more than 90.2% 5-fluorouracil and 77.6% heparin at pH 5.2 within 48 h, while release remained below 60% at physiological pH (Pandya et al., 2025).

In a subset of platforms, acidic pH did more than accelerate release; it actively changed the carrier’s surface state. Metal–organic framework@polymer nanoparticles maintained a negative surface during circulation but exposed a positively charged metal–organic framework core after acid-mediated degradation of the polymer shell (Hu et al., 2022). Programmed PEOz-b-PSD/poly (amidoamine) nanoparticles underwent protonation-driven conversion from larger negatively charged particles to positively charged ultrafine particles (Jia et al., 2020). Related systems used acid-induced charge conversion, shielding removal, cationization, particle-size transition, or core swelling, including polyacrylated hyaluronic acid-coated protein cages, charge-reversal nanoplatforms, azobenzene-containing covalent organic framework nanocarriers carrying doxorubicin, and polyethylene glycol-block-poly (aspartic acid) micelles with piperidine-based charge switching (Shi et al., 2020; Tan et al., 2024; Wang et al., 2024a; Zhang N. X. et al., 2021). This charge-conversion strategy is mechanistically important because it links tumor acidity to a programmed change in carrier interaction, penetration, or release behavior.

### Redox and glutathione responsiveness for intracellular activation

Redox-responsive systems were primarily engineered to release cargo under reductive tumor-associated or intracellular conditions, commonly modeled with glutathione (GSH) or dithiothreitol. These platforms used disulfide cleavage, coordination disruption, metal oxide reduction, nanogel dissociation, or prodrug activation to convert stable carriers into release-active systems. Representative examples included triblock copolymer micelles, dextran prodrug vesicles, disulfide magnetic nanocarriers, hyaluronic acid–disulfide–celastrol micelles, redox-sensitive polymeric nanoparticles, xylan-curcumin prodrugs, dopamine-modified hyaluronic acid/copper (II)/6-mercaptopurine coordination particles, luteinizing hormone-releasing hormone-disulfide crosslinked micelles, magnetic mesoporous silica, and gemcitabine-conjugated hyaluronic acid–disulfide nanoprodrugs (Augustine et al., 2020; Curcio et al., 2020; Ehsanimehr et al., 2025; Fu et al., 2025; Gupta et al., 2022; Gupta et al., 2024; Sauraj et al., 2020; Tao and Yin, 2020; Xiao et al., 2021; Zhang D. et al., 2024; Zhang F. et al., 2023).

The clearest contribution of redox engineering is kinetic separation between extracellular retention and intracellular release. The approximately 59 nm nanoparticles reported by Gupta et al. released about 98% doxorubicin and 93% curcumin in 10 mM GSH, while release remained low under physiological conditions (Gupta et al., 2022). Disulfide-containing poly (lactic acid)-block-polyethylene glycol nanoparticles remained stable for up to 3 weeks and released more drug in reducing media: pirarubicin release increased from 20.5% ± 1.0 at pH 7.4%–40.1% ± 0.4 in dithiothreitol, while salinomycin release increased from 36.2% ± 1.7%–51.5% ± 1.7 (Gupta et al., 2024). The unimolecular micelles reported by Li and Liu showed 62.0% drug content, a hydrodynamic diameter of 122 nm, 60.9% GSH-triggered cumulative release within 85 h, and only 3.2% premature leakage (Li and Liu, 2022). Dopamine-modified hyaluronic acid/copper (II)/6-mercaptopurine particles used Cu^2+^ coordination and redox-triggered release, with a 173.5 nm particle size, 49.5 mg/g drug loading, 70.18% encapsulation efficiency, greater than 94% release within 2 h in GSH-containing media, and only 15% release over 24 h without GSH (Tao and Yin, 2020). GSH-responsive magnetic mesoporous silica released up to 43% of loaded doxorubicin under reducing conditions (Zhang D. et al., 2024). Together, these examples show how redox-sensitive bonds and coordination motifs provide a material basis for controlled intracellular activation.

### Multi-stimuli and externally activated delivery platforms

A major development across the reviewed studies was the integration of more than one activation cue into the same platform. Multi-stimuli systems were designed to coordinate release through pH/redox, pH/temperature, pH/hypoxia, enzyme/redox, reactive oxygen species-responsive, photothermal, photodynamic, or ultrasound-triggered mechanisms (Augustine et al., 2020; Chen et al., 2026; Du et al., 2020; Jahanban-Esfahlan et al., 2020; Li et al., 2021; Maiti et al., 2025; Motamedi et al., 2020; Song et al., 2023; Wang L. et al., 2025; Yang et al., 2022; Zhang Y. P. et al., 2025). Compared with single-trigger systems, these designs more explicitly couple extracellular priming, intracellular activation, or externally controlled release with carrier transformation. This does not by itself establish therapeutic superiority, but it clarifies how multi-step responsiveness can be engineered into the delivery process.

Several systems illustrate this layered design logic. Triblock copolymer micelles combined pH, temperature, and redox-sensitive behavior, forming approximately 200 nm micelles with about 50% doxorubicin encapsulation (Augustine et al., 2020). Keratin–polyethylene glycol micelles combined pH, GSH, trypsin responsiveness, charge reversibility, and physiological stability (Du et al., 2020). Hollow nanocapsules integrated pH and thermal responsiveness, with a lower critical solution temperature of 38 °C–40 °C, 62% drug-loading efficiency, and 53% encapsulation efficiency (Jahanban-Esfahlan et al., 2020). Star-shaped poly (ε-caprolactone)-graft-[poly (2-dimethylaminoethyl methacrylate)-block-poly (N-isopropylacrylamide)] terpolymers achieved 36.9% ± 4.1% methotrexate loading and released more drug under pH 5 °C and 41 °C conditions (Motamedi et al., 2020). Hybrid nanoparticles with a calcium phosphate shell and disulfide-crosslinked interlayer limited leakage at physiological pH, while acidic and hypoxic conditions promoted shell dissolution and network breakage (Li et al., 2021). Dual-pH and pH/redox systems further refined this approach by coordinating sequential release from different carrier compartments or bonds, including mesoporous silica/polyacrylic acid/pH-sensitive lipid/F56 peptide systems carrying paclitaxel and arsenic trioxide and folic-acid-modified, bovine serum albumin-cloaked hollow mesoporous silica nanoparticles (Zhang B. B. et al., 2021; Zhang and Liang, 2024).

Externally activated systems added temporal control through light or ultrasound. Copper sulfide@mesoporous silica@polydopamine systems combined pH and photothermal-responsive release, reaching 67.1% cumulative release at pH 6.5 by day 10 and increasing photothermal conversion efficiency from 42.7% for copper sulfide@mesoporous silica to 59.9% after polydopamine coating (Chen et al., 2026). Polyethylene glycol-PABI/gold nanocluster micelles of approximately 120 nm combined acidic release with photothermal stability under repeated near-infrared exposure (Husni et al., 2023). Carbon dot–metal organic gels achieved 72.9% loading capacity and used 450 nm visible light to reduce Fe^3+^ to Fe^2+^, enabling hydroxyl-radical generation from H_2_O_2_ while maintaining pH-responsive release (Maiti et al., 2025). Hypoxia-responsive polymersomes released more than 90% doxorubicin within 12 h at 2% oxygen, compared with approximately 30% under normoxic conditions (Mamnoon et al., 2020). Hypoxia-cleavable Exatecan liposomes combined pH-sensitive lipids, reductive stress, IR808-mediated reactive oxygen species generation, aggravated hypoxia, and azobenzene cleavage (Wang L. et al., 2025). Ultrasound-responsive conjugated polymer nanoparticles generated reactive oxygen species after acoustic activation, which cleaved internal junctions and released camptothecin (Zhang Y. P. et al., 2025). These systems show how endogenous stress signals and externally applied energy can be engineered as inputs for controlled release and activation.

### Enzyme, matrix, and barrier-responsive mechanisms

Enzyme and matrix-responsive platforms used tumor-associated proteases, hyaluronidase, proteinase K, or extracellular matrix conditions as triggers for release, deshielding, disassembly, or local barrier modification. Chitosan/gold nanoparticle–gelatin nanogels released doxorubicin through an enzyme-responsive mechanism, with size increasing from approximately 83 nm for chitosan/gold nanoparticles to 119.3 nm after gelatin incorporation and doxorubicin loading of approximately 56% (Aslzad et al., 2023). Branched polyethyleneimine-disulfide-platinum/hyaluronidase@calcium phosphate nanocomplexes, with an optimized size of 143 ± 14 nm, combined tumor-responsive hyaluronidase release with intracellular GSH-triggered cisplatin release through disulfide cleavage (Jia et al., 2022). Methacryloylated hyaluronic acid-coated mesoporous silica nanoparticles were designed to disassemble in response to tumor-abundant hyaluronidase (Liu et al., 2025). Gelatin–concanavalin A nanoparticles used matrix metalloproteinase-triggered cisplatin release (Vaghasiya et al., 2021), while micelles incorporating D-α-tocopheryl polyethylene glycol 3,350, matrix metalloproteinase-2-cleavable PVGLIG-doxorubicin, and folic acid-disulfide-doxorubicin modules used matrix metalloproteinase-2 cleavage to remove shielding before GSH-mediated intracellular doxorubicin release (Wan et al., 2024). Poly (methacrylic acid) nanohydrogels provided pH-responsive chloroquine release and enzyme-facilitated methotrexate release under proteinase K exposure (Rashidzadeh et al., 2023). Acid-responsive bromelain/doxorubicin nanogels co-encapsulated bromelain and acid-labile doxorubicin, using bromelain release to degrade collagen-rich extracellular matrix while concurrently releasing doxorubicin (Wang L. J. et al., 2025).

This group of studies also highlights a broader principle: delivery barriers can be treated as engineering variables. Liposomal nanoparticles of approximately 126, 164, and 234 nm did not diffuse beyond 100 μm from the nearest blood vessel, illustrating a material transport limitation for conventional liposomal delivery (Samson et al., 2020). Protein cages protected by polyacrylated hyaluronic acid coatings underwent acid- and hyaluronidase-triggered deshielding, size reduction, and charge reversal (Shi et al., 2020). Nanoparticle-loaded macrophages were evaluated in a three-dimensional tumor-microenvironment-on-a-chip model as a cell-mediated transport strategy through tumor-like matrices (Wang et al., 2020). Stimuli-responsive transcytosis systems were also presented as a design principle for improving penetration beyond passive delivery constraints (Wang et al., 2024b). These examples connect the section’s mechanistic focus to the broader precision-delivery challenge: stimulus-responsive behavior can regulate not only release but also carrier access, penetration, and transport mode.

### Carrier architecture, stability, and leakage control

The effectiveness of stimuli-responsive delivery depends on the underlying carrier architecture. Across the reviewed studies, controlled delivery was supported by core–shell design, hollow or porous structures, crosslinked micelles, gatekeepers, polymer brushes, hydrogels, protein cages, coordination networks, prodrug assemblies, and mesoporous frameworks. Alcohol-free lysozyme–hyaluronan self-assembly produced homogeneous nanoparticles with a size of 126.1 nm and a polydispersity index of 0.1 (Li et al., 2024). Core-crosslinked micelles decreased in size from 96.43 nm in non-crosslinked micelles to 72.63 nm after crosslinking and achieved 7.32% SN-38 loading with 86.23% encapsulation efficiency (Gebrie et al., 2022). Artificial intelligence-guided lignin@graphene oxide@zeolitic imidazolate framework-8 formulation used an extreme-gradient-boosting model trained on 500 formulation–property pairs, with R^2^ values of 0.86–0.89 for predicting drug loading, encapsulation efficiency, and release rates (Gorish et al., 2026). Carboxyl-functionalized mesoporous silica/hyperbranched polymer platforms incorporating bicalutamide and folic acid achieved a reported loading capacity of 268.60% after carboxyl alteration (Feng et al., 2023). UiO-66(Zr)-based carriers showed 62% binding efficiency, 5-fluorouracil encapsulation of 137.1 mg/g for UiO-66(Zr)-NH_2_ and 45.0 mg/g for UiO-66(Zr)-His, and diffusion-controlled release with R^2^ > 0.9 (Migasova et al., 2025). Porous silica nanoparticles offered tunable sizes of 42–178 nm, pores of 1.1–2.7 nm, 22% w/w doxorubicin loading, and a fourfold increase in weakly acidic release (Newham et al., 2021).

Leakage control was a recurring architectural priority because premature cargo loss would weaken the logic of stimulus-responsive precision delivery. MnO_2_-capped hollow polydopamine spheres used GSH-sensitive caps to prevent premature release (Miao et al., 2022). Folic-acid-modified, bovine serum albumin-cloaked hollow mesoporous silica used bovine serum albumin as a gatekeeper, with pH/redox cleavage of imine and disulfide bonds enabling release (Zhang and Liang, 2024). Biodegradable hollow mesoporous organosilica nanoparticles incorporated acetal-containing pH-cleavable bridges into hollow mesoporous organosilica frameworks, while π–π interactions reduced premature leakage and weak acidity promoted biodegradation (Yang et al., 2020). Amine-functionalized cellulose nanogels combined porosity, permeability, colloidal stability, pH-responsive phosphate-buffered saline absorption, and 86.7% doxorubicin-loading efficiency (Raj and Sundaramoorthy, 2025). These architectural examples show that stimulus responsiveness is not an isolated chemical feature; it depends on carrier structure, cargo-retention strategy, and the ability to transition from a stable state to a release-active state under defined conditions.

Overall, the reviewed platforms show that precision in stimuli-responsive cancer delivery arises from the deliberate coupling of material design with defined activation cues. Acidic pH, reductive GSH, tumor-associated enzymes, reactive oxygen species, hypoxia, heat, light, ultrasound, and matrix conditions function as programmable inputs that alter carrier charge, stability, permeability, degradation, disassembly, or release kinetics. The strongest examples link these stimulus-triggered transitions to measurable material performance, including loading capacity, encapsulation efficiency, leakage suppression, particle-size transformation, controlled degradation, photothermal conversion, and release profiles. This mechanistic framework explains how bioengineered platforms can be designed for precision activation without relying on unsupported claims about downstream therapeutic superiority. Table 3 summarizes the major functional mechanisms, activation cues, platform responses, and representative formulation data discussed in this section.

**TABLE 3:** Engineered functional mechanisms linking tumor-associated stimuli to precision delivery behavior.

Engineered functional mechanism	Stimulus or activation cue	Platform response supporting precision delivery	Key technical/formulation data appropriate for this section	Representative references
Acid-triggered release and degradable carrier activation	Acidic tumor, endosomal, or lysosomal pH	Protonation, swelling, framework dissolution, shell degradation, hydrogel destabilization, or micellar dissociation increases release under acidic conditions while limiting premature release at physiological pH	Hyaluronic acid/carboxymethyl dextran/gelatin methacryloyl nanogels: 80%–90% entrapment, 100–200 nm size, and sustained release at pH 5.0; hollow silica: 292 ± 14 nm inner void, 11.8 nm pores, 0.509 g/g loading, and 87.5% release at pH 5.0; ionic-liquid hydrogel: >90.2% 5-fluorouracil and 77.6% heparin release at pH 5.2 within 48 h, with <60% release at physiological pH	(Abd Al-Jabbar et al., 2022; Aghaz et al., 2023; Bhattacharya et al., 2024; Jin et al., 2021b; Kazemi et al., 2025b; Ozkahraman et al., 2026; Pandya et al., 2025; Xu et al., 2022)
pH-induced charge conversion and deshielding	Mild extracellular acidity or stronger intracellular acidity	Surface charge reversal, cationization, shielding removal, particle-size transition, or core swelling converts circulation-stable carriers into release-active or interaction-ready systems	Sericin nanoparticles shifted from negative charge at physiological pH toward positive charge in acidic media; metal–organic framework@polymer particles exposed a positive metal–organic framework core after acid-mediated shell degradation; polyethylene glycol-block-poly (aspartic acid) micelles used piperidine protonation for charge reversal and core swelling	(Aghaz et al., 2023; Hu et al., 2022; Jia et al., 2020; Shi et al., 2020; Tan et al., 2024; Wang et al., 2024a; Zhang et al., 2021b)
Redox- and GSH-responsive intracellular activation	Reductive environments, commonly GSH or dithiothreitol	Disulfide cleavage, coordination disruption, metal oxide reduction, nanogel dissociation, or prodrug activation releases cargo preferentially under reductive tumor or intracellular conditions	Approximately 59 nm nanoparticles released approximately 98% doxorubicin and 93% curcumin in 10 mM GSH; unimolecular micelles showed 62.0% drug content, 122 nm size, 60.9% GSH-triggered release within 85 h, and 3.2% premature leakage; dopamine-modified hyaluronic acid/copper (II)/6-mercaptopurine particles showed 173.5 nm size, 49.5 mg/g loading, 70.18% encapsulation, and >94% release within 2 h in GSH.	(Curcio et al., 2020; Gupta et al., 2022; Gupta et al., 2024; Li and Liu, 2022; Sauraj et al., 2020; Tao and Yin, 2020; Xiao et al., 2021; Zhang et al., 2024a; Zhang et al., 2023a)
Enzyme- and matrix-responsive disassembly	Hyaluronidase, matrix metalloproteinases, proteinase K, or collagen-rich extracellular matrix	Enzyme-triggered coating removal, carrier disassembly, protease-sensitive release, or matrix degradation links delivery to tumor-associated enzymatic activity and local transport barriers	Gelatin nanogel: size increased from approximately 83 nm–119.3 nm after gelatin incorporation, with approximately 56% doxorubicin loading; hyaluronidase@calcium phosphate nanocomplexes: 143 ± 14 nm; poly (methacrylic acid) nanohydrogels: <50 nm size, 4.99% methotrexate loading, 25.01% chloroquine loading, and enzyme-facilitated methotrexate release	(Aslzad et al., 2023; Jia et al., 2022; Liu et al., 2025; Rashidzadeh et al., 2023; Vaghasiya et al., 2021; Wan et al., 2024; Wang et al., 2025b)
Integrated multi-stimuli sequential release	Combined pH, redox, temperature, hypoxia, enzyme, or shell/core cues	Multiple triggers coordinate staged activation, such as extracellular priming followed by intracellular release, or shell dissolution followed by network cleavage	Triblock copolymer micelles: approximately 200 nm and approximately 50% doxorubicin encapsulation; hollow nanocapsules: lower critical solution temperature of 38 °C–40 °C, 62% loading efficiency, and 53% encapsulation efficiency; hybrid nanoparticles with a calcium phosphate shell and disulfide-crosslinked interlayer: 157.9 ± 3.9 nm; star terpolymer: 36.9% ± 4.1% methotrexate loading	(Augustine et al., 2020; Du et al., 2020; Jahanban-Esfahlan et al., 2020; Li et al., 2021; Motamedi et al., 2020; Zhang et al., 2021a; Zhang and Liang, 2024)
Externally activated reactive oxygen species, photothermal, photodynamic, hypoxia, or ultrasound mechanisms	Reactive oxygen species, hypoxia, near-infrared or visible light, photothermal heating, photodynamic activation, or ultrasound	Internal stress signals or externally applied energy trigger bond cleavage, reactive oxygen species generation, photothermal conversion, hypoxia-sensitive release, or sonodynamic drug liberation	Copper sulfide@mesoporous silica@polydopamine systems: 67.1% cumulative release at pH 6.5 by day 10, with photothermal conversion increasing from 42.7% to 59.9% after polydopamine coating; carbon dot–metal organic gel: 72.9% loading and 450 nm light activation; hypoxia-responsive polymersomes: >90% doxorubicin release at 2% O_2_ within 12 h *versus* approximately 30% under normoxia	(Ayyanaar et al., 2020; Chen et al., 2026; Cheng et al., 2023; Husni et al., 2023; Maiti et al., 2025; Mamnoon et al., 2020; Wang et al., 2025a; Zhang et al., 2025b)
Architecture-driven stability, loading, and leakage control	Carrier structure, crosslinking, gating, porosity, or coating design	Core–shell organization, hollow or porous structures, crosslinking, gatekeepers, polymer brushes, and diffusion-controlled frameworks retain cargo before stimulus exposure and regulate release afterward	Core-crosslinked micelles: 96.43 nm reduced to 72.63 nm after crosslinking, 7.32% SN-38 loading, and 86.23% encapsulation; UiO-66 carriers: 62% binding efficiency, 137.1 and 45.0 mg/g 5-fluorouracil encapsulation, and diffusion-controlled release with *R* ^2^ > 0.9; porous silica: 42–178 nm size, 1.1–2.7 nm pores, and 22% w/w doxorubicin loading	(Feng et al., 2023; Gebrie et al., 2022; Gorish et al., 2026; Miao et al., 2022; Migasova et al., 2025; Newham et al., 2021; Raj and Sundaramoorthy, 2025; Yang et al., 2020)
Barrier-responsive penetration and transport logic	Limited diffusion, dense matrix, deshielding cues, or active carrier transport	Programmable size change, charge conversion, matrix remodeling, or cell-mediated transport addresses penetration constraints that passive nanocarriers alone may not overcome	Liposomes of approximately 126, 164, and 234 nm did not diffuse beyond 100 μm from the nearest vessels; polyacrylated hyaluronic acid-coated protein cages underwent acid/enzyme-triggered deshielding, size reduction, and positive charge conversion; macrophage-loaded nanoparticles were evaluated as cell-mediated carriers in a three-dimensional tumor-microenvironment-on-a-chip model	(Samson et al., 2020; Shi et al., 2020; Wang et al., 2020; Wang et al., 2024b)

## Preclinical biological validation

### Cellular activity, stimulus-linked uptake, and tumor-selective cytotoxicity

Preclinical biological validation of stimuli-responsive bioengineered platforms was primarily established through cancer-cell viability, cytotoxicity, uptake, apoptosis, migration, invasion, colony-formation, spheroid, and cell-cycle assays. These assays provide the first level of evidence that pH, redox, enzyme, hypoxia, reactive oxygen species (ROS), thermo, light, or ultrasound-responsive designs can convert tumor-associated signals into measurable therapeutic effects. Breast cancer models were the most extensively represented, including MCF-7, MDA-MB-231, 4T1, SKBR3, SUM-149, MCF-7/ADR, and triple-negative breast cancer systems. Across these models, responsive platforms produced stronger biological activity than free drugs, single-agent treatments, or nontargeted controls, with enhanced uptake, increased apoptosis, reduced migration or invasion, and improved selectivity toward malignant cells (Abd Al-Jabbar et al., 2022; Aghaz et al., 2023; Arvejeh et al., 2025; Feng et al., 2023; Hu et al., 2022; Karimifard et al., 2022; Mamnoon et al., 2020; Nie et al., 2023; Rahmani et al., 2021; Xiao et al., 2021). Several studies provided quantitative support for these effects. Aptamer-functionalized, doxorubicin-loaded pollen-like silica particles reduced viable MCF-7 cells to 4.2%, while showing minimal uptake in normal cells and near-complete viability before drug loading (Jin R. R. et al., 2021). An SN-38-loaded responsive nanocarrier showed half-maximal inhibitory concentration (IC_50_) values of 1.8 μg/mL in MDA-MB-231 cells and 1.7 μg/mL in 4T1 cells, with apoptotic ratios of 27.8 and 32.8, respectively (Nie et al., 2023). Doxorubicin/conferone micelles achieved 100% internalization within 30 min and induced apoptosis in more than 98% of MDA-MB-231 cells at an IC_50_ of 0.259 μg/mL (Rahmani et al., 2021). Together, these findings show that the biological value of stimulus-responsive design was strongest when enhanced intracellular access was paired with apoptosis induction, cytotoxic potency, or reduced effects on nonmalignant controls.

Validation was not limited to breast cancer. Lung, cervical, colorectal, hepatic, prostate, gastric, and glioblastoma models were used to test whether responsive behavior translated into measurable therapeutic effects across different tumor contexts. In A549 and HeLa S3 cells, iron oxide/oleic acid-chitosan nanoparticles carrying 5-fluorouracil inhibited colony formation and produced IC_50_ values of 12.9 μg/mL and 23 μg/mL after 24 h (Ayyanaar et al., 2020). In HCT116 colorectal cancer cells, bosutinib-loaded nanogels reduced cell survival by 70%–80% at higher doses, reduced migration, and showed antiangiogenic activity in a chick chorioallantoic membrane model (Bhattacharya et al., 2024). In SW480 colon cancer cells, methotrexate/chloroquine nanohydrogels reduced viability to 29% while maintaining approximately 100% human foreskin fibroblast 2 cell viability and less than 5% red-blood-cell hemolysis (Rashidzadeh et al., 2023). Enzyme-responsive cisplatin delivery in A549 cells enhanced ROS generation, apoptosis, and S/G_2_-M cell-cycle arrest in the presence of matrix metalloproteinase-2 (Vaghasiya et al., 2021). These results extend the evidence base beyond short-term cytotoxicity toward functional endpoints associated with tumor progression and treatment response.

### Penetration, three-dimensional models, and tumor microenvironment barriers

A central question for precision cancer therapy is whether responsive platforms can reach tumor compartments that are poorly served by conventional delivery. Accordingly, many studies evaluated intracellular uptake, nuclear localization, spheroid access, stromal remodeling, and microenvironment-dependent penetration. Confocal microscopy, fluorescence microscopy, flow cytometry, fluorescein isothiocyanate/4′,6-diamidino-2-phenylindole staining, and related tracking approaches confirmed enhanced uptake or internalization in multiple systems, including rapid uptake under acidic conditions, cationic fluorescent nanogel internalization at 100 μg/mL within 5 min, reduced nonspecific uptake by RAW264.7 macrophage-like cells, and greater accumulation in patient-derived glioblastoma cells than in neural progenitor cells (Aghaz et al., 2023; Fattahi et al., 2024; González-Urías et al., 2021; Huang et al., 2022; Newham et al., 2021). Receptor-mediated uptake was also demonstrated in triple-negative breast cancer models treated with luteinizing hormone-releasing hormone-targeted dendritic cholic acid micelles (Xiao et al., 2021).

**FIGURE 4:**
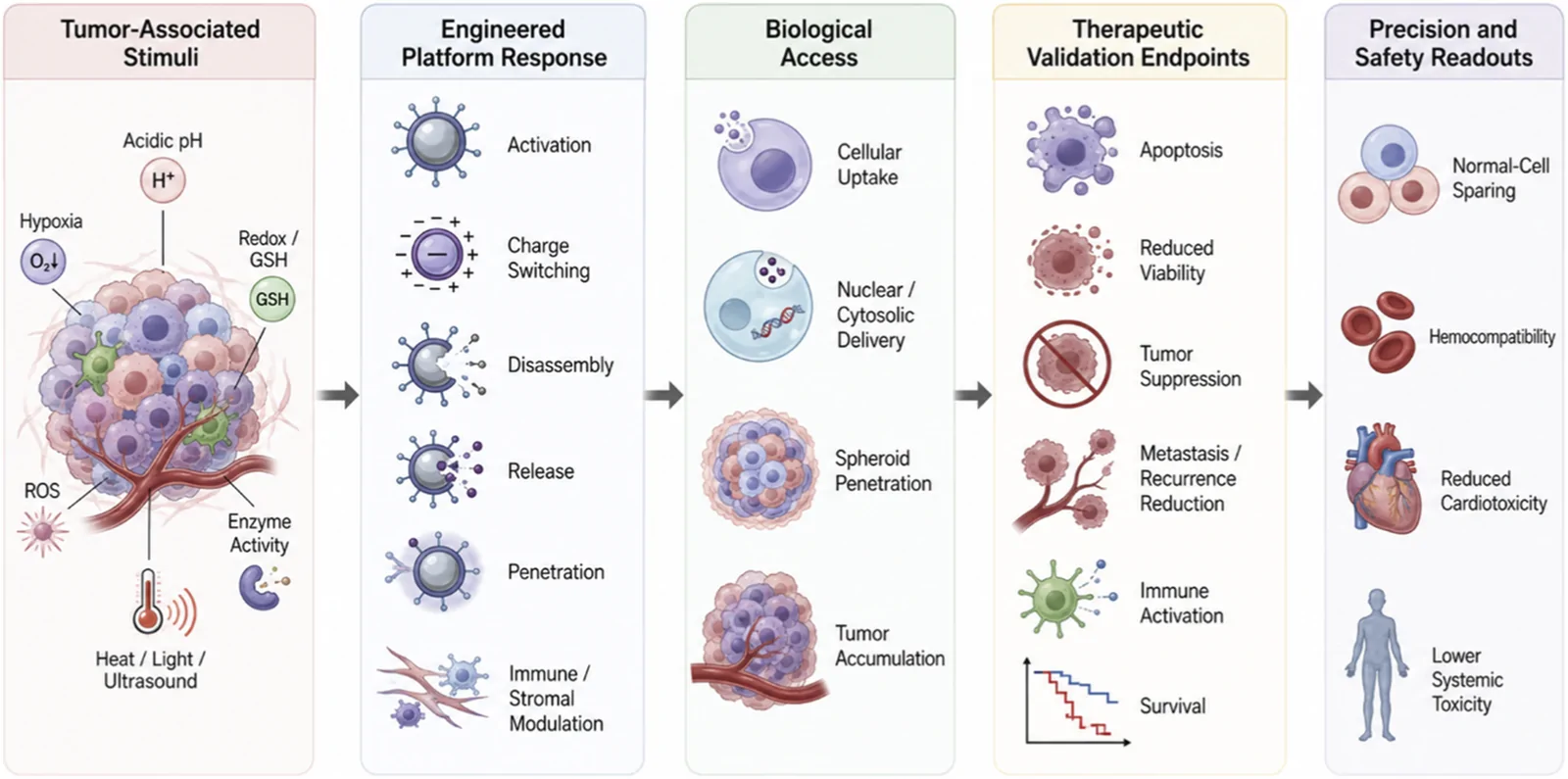
Preclinical Biological Validation Framework for Stimuli-Responsive Precision Cancer Platforms. This figure demonstrates a left-to-right preclinical validation workflow for stimuli-responsive cancer platforms. Tumor-associated and external triggers, including acidic pH, redox/GSH, hypoxia, ROS, enzymes, heat, light, and ultrasound, initiate platform activation. Biological performance is then evaluated across escalating models, from cellular assays to 3D tumor microenvironment systems and *in vivo* tumor models. Representative readouts assess delivery, penetration, tumor accumulation, antitumor activity, metastasis or recurrence reduction, survival, stromal or immune remodeling, and safety. Together, these validation tiers generate precision-relevant biological evidence, including tumor-selective activity, improved access to difficult tumor regions, enhanced efficacy, and reduced off-target toxicity (Prepared with the assistance of Gemini’s NotebookLM Pro and ChatGPT Plus).

Three-dimensional and tissue-level models strengthened the biological relevance of these findings by testing delivery under more restrictive tumor-like conditions. Hypoxia-responsive polymersomes showed greater cytotoxicity in hypoxic MCF-7 spheroids than in normoxic spheroids (Mamnoon et al., 2020), UiO-66(Zr)-His carrying 5-fluorouracil reduced U87MG spheroid formation (Migasova et al., 2025), and the charge-reversal nanoplatform reported by Tan et al. inhibited proliferation, migration, and three-dimensional tumorsphere growth while also improving tumor delivery and penetration *in vivo* (Tan et al., 2024). The importance of penetration as a distinct validation endpoint was underscored by spatial mapping in intact transparent tumor tissues. Liposomal nanoparticles did not diffuse more than 100 μm from the nearest blood vessel, whereas hypoxia-inducible factor 1 alpha-positive cells extended from 62 to approximately 460 μm and cluster of differentiation 44-positive cells reached approximately 300 μm from vessels (Samson et al., 2020). This evidence clarifies why bulk tumor accumulation alone is insufficient for validating precision delivery: hypoxic, clonogenic, stromal, and poorly perfused regions may remain inaccessible unless the platform addresses tumor architecture or transport barriers. In this context, drug-loaded macrophages migrated toward and infiltrated tumor spheroids in a tumor-microenvironment-on-a-chip model and produced greater tumor-cell cytotoxicity than nanoparticles alone (Wang et al., 2020), while bromelain-containing nanogels degraded collagen-rich extracellular matrix, improved doxorubicin penetration and distribution, and reduced osteosarcoma tumor burden *in vivo* (Wang L. J. et al., 2025). The validation logic connecting tumor-associated stimuli to platform response, biological access, therapeutic endpoints, and safety readouts is summarized conceptually in Figure 4.

### *In vivo* tumor suppression, metastasis, recurrence, and survival

*In vivo* validation provided the strongest evidence that stimulus-responsive behavior could translate into therapeutic benefit beyond isolated cellular assays. Murine models were used to assess tumor-growth inhibition, biodistribution, tumor accumulation, metastasis, recurrence, survival, and comparative efficacy against free drugs, monotherapies, nonresponsive carriers, or clinical comparators. In breast cancer and related tumor-bearing models, responsive systems reduced tumor size, improved tumor accumulation, inhibited lung metastasis, or outperformed free drugs and nontargeted formulations (Arvejeh et al., 2025; Chen et al., 2026; Guo et al., 2020; Hu et al., 2022; Jiang et al., 2021; Shi et al., 2020; Song et al., 2023; Taghdisi et al., 2020; Xiao et al., 2021). Quantitative examples included an 80% lower recurrence rate after tumor resection compared with metformin alone (Chen et al., 2026) and approximately 100% tumor regression in BALB/c mice treated with dual-loaded redox-responsive nanoparticles, without systemic toxicity in vital organs compared with free drugs (Gupta et al., 2024). Additional *in vivo* outcomes included 60.1% colorectal tumor-growth inhibition by 5-fluorouracil/curcumin-loaded lysozyme–hyaluronan nanoparticles (Li et al., 2024), 75.12% tumor inhibition in H22 tumor-bearing mice (Fu et al., 2025), 88.0% tumor inhibition by a doxorubicin/Bcl-2 small interfering RNA co-delivery system (Yan et al., 2020), 87.29% tumor inhibition by irinotecan/imiquimod micelles in CT26 tumor-bearing mice (Yan et al., 2023), and 76.98% ± 9.09% tumor suppression by ultrasound-triggered ROS-responsive nanoparticles in hormonal mouse breast cancer models without cardiotoxicity or other systemic side effects (Zhang Y. P. et al., 2025).

Several platforms were evaluated against more advanced disease-relevant endpoints, including metastatic spread, recurrence, survival, and treatment resistance. In 4T1 tumor-bearing mice, ROS-responsive chemoimmunotherapy reduced primary tumor burden and lung metastasis (Song et al., 2023), while ROS-responsive ovarian cancer micelles inhibited tumor growth, invasion, angiogenesis, recurrence, and metastasis (Zhang L. et al., 2025). In glioblastoma, combined micelle and radiotherapy treatment reduced U251 cell viability to 64.0% and proliferation capacity to 16.3%, produced substantial accumulation and tissue injury in xenograft tumors, and extended median survival from 26 to 56 days (Zhang et al., 2022). In two preclinical tumor models, 4PSC/paclitaxel nanomicelles enhanced paclitaxel activity and inhibited tumor proliferation, invasiveness, and chemotherapy-induced resistance without systemic toxicity (Zhou et al., 2023). These findings are central to the manuscript’s precision-therapy logic because they show that responsive platforms were not evaluated solely as drug carriers but as systems designed to improve biological control of tumor growth, dissemination, recurrence, resistance, or survival.

### Immune, stromal, and safety validation

A substantial subset of platforms was validated through immune activation, macrophage polarization, dendritic-cell maturation, cytokine production, T-cell infiltration, stromal remodeling, hemocompatibility, cardiotoxicity monitoring, and systemic-toxicity assessment. These endpoints are especially relevant to precision cancer therapy because they evaluate whether a responsive platform can reshape the tumor microenvironment or improve tolerability while maintaining antitumor efficacy. Chemoimmunotherapy-oriented systems induced immunogenic cell death, increased effector immune-cell infiltration, reduced immunosuppressive cell populations, and improved antitumor immune activity (Li et al., 2025; Phung et al., 2023; Song et al., 2023; Yan et al., 2023; Zhang G. Z. et al., 2023; Zhang L. et al., 2024). In orthotopic lung cancer, paclitaxel-loaded anti–programmed cell death protein 1 antibody-containing ionic-liquid stealth nanoparticles increased tumor necrosis factor alpha and interferon gamma, promoted cluster of differentiation 8-positive T-cell recruitment, restored T-cell immunocidal function, induced immunogenic cell death, and supported dendritic-cell maturation (Li et al., 2025). In CT26 tumor-bearing mice, irinotecan/imiquimod micelles increased intratumoral SN-38 and imiquimod concentrations by 9.39-fold and 3.44-fold, respectively, while promoting dendritic-cell maturation, cluster of differentiation 8-positive T-cell infiltration, and macrophage secretion of tumor-killing cytokines (Yan et al., 2023). In pancreatic and ovarian cancer models, responsive platforms shifted macrophages from M2 toward M1 phenotypes, increased cytotoxic T-lymphocyte density, remodeled immunosuppressive microenvironments, and inhibited invasion, angiogenesis, recurrence, or metastasis (Zhang G. Z. et al., 2023; Zhang L. et al., 2024; Zhang L. et al., 2025). Other platforms directly addressed stromal or extracellular matrix barriers, including Gem/Sul-NP-mediated enhancement of gemcitabine uptake and cluster of differentiation 8-positive, interferon gamma-positive T-cell infiltration when combined with anti–programmed cell death protein 1 therapy (Jiang et al., 2025), *Camellia oleifera* protein nanoparticle-mediated reduction of tumor interstitial fluid pressure and solid stress with increased tumor drug accumulation and prolonged survival (Qian et al., 2020), and bromelain nanogel-mediated collagen degradation with improved doxorubicin penetration and distribution (Wang L. J. et al., 2025).

Safety validation was addressed through normal-cell controls, hemolysis assays, zebrafish testing, organ-toxicity evaluation, cardiotoxicity monitoring, and systemic-toxicity observation. Representative findings included reduced toxicity toward human umbilical vein endothelial cells, HaCaT cells, L02 cells, L929 cells, 3T3 cells, peripheral blood mononuclear cells, and human foreskin fibroblast 2 cells (Abd Al-Jabbar et al., 2022; Du et al., 2020; Li and Liu, 2022; Maiti et al., 2025; Mathes et al., 2024; Pandya et al., 2025; Rashidzadeh et al., 2023). Keratin-based micelles showed high toxicity toward A549 cells with minimal effects on normal cells and no hemolysis (Du et al., 2020), while methotrexate/chloroquine nanohydrogels showed approximately 100% human foreskin fibroblast 2 cell viability and less than 5% hemolysis (Rashidzadeh et al., 2023). Zebrafish embryo testing of ruthenium-loaded nanogels produced median lethal dose values of 1,185.93 and 823.03 μM (Pragti et al., 2023). *In vivo* safety outcomes included reduced doxorubicin-associated cardiotoxicity, favorable biosafety, lack of observable systemic toxicity, and minimized side effects compared with free drugs, Taxol, or nonspecific drug combinations (Jia et al., 2020; Miao et al., 2020; Shi et al., 2020; Wang L. et al., 2025; Wang L. J. et al., 2025; Xiao et al., 2021; Zhang Y. P. et al., 2025). Collectively, the most persuasive preclinical evidence pairs antitumor efficacy with cancer-cell selectivity, improved penetration, microenvironment modulation, or reduced off-target toxicity, thereby supporting the rationale for stimuli-responsive bioengineered platforms as precision-oriented therapeutic systems. Representative validation patterns are summarized in Table 4.

**TABLE 4:** Preclinical biological validation patterns for stimuli-responsive bioengineered platforms in precision cancer therapy.

Preclinical biological validation focus	Stimuli-responsive precision feature being validated	Representative models and endpoints	Key biological outcomes and reviewer-relevant data	Representative references
Stimulus-enhanced cancer-cell killing	pH-, redox-, enzyme-, hypoxia-, ROS-, or thermo-responsive activation linked to stronger tumor-cell cytotoxicity	MCF-7, MDA-MB-231, 4T1, A549, HeLa, HCT116, and SW480 cells; viability, 3-(4,5-dimethylthiazol-2-yl)-2,5-diphenyltetrazolium bromide assay, Cell Counting Kit-8 assay, apoptosis, cell-cycle, and colony-formation assays	Stimulus-responsive systems frequently outperformed free drugs, monotherapies, or nontargeted controls. Key results included 4.2% viable MCF-7 cells after aptamer-functionalized, doxorubicin-loaded pollen-like silica particle treatment; IC_50_ values of 1.8 μg/mL and 1.7 μg/mL in MDA-MB-231 and 4T1 cells for an SN-38-loaded responsive nanocarrier; SW480 viability reduced to 29% by methotrexate/chloroquine nanohydrogels; and matrix metalloproteinase-2-enhanced ROS generation, apoptosis, and S/G_2_-M arrest in A549 cells	(Ayyanaar et al., 2020; Jin et al., 2021b; Nie et al., 2023; Rahmani et al., 2021; Rashidzadeh et al., 2023; Vaghasiya et al., 2021)
Selective uptake and intracellular delivery	Ligand targeting, charge reversal, endocytosis enhancement, or stimulus-triggered intracellular or nuclear release	Fluorescence microscopy, confocal microscopy, flow cytometry, fluorescein isothiocyanate/4′,6-diamidino-2-phenylindole staining; cancer cells, macrophage-like cells, and normal-cell controls	Targeted or responsive systems improved intracellular access while reducing nonspecific uptake where tested. Reported findings included enhanced uptake at acidic pH, cationic nanogel internalization at 100 μg/mL within 5 min, reduced uptake by RAW264.7 macrophage-like cells, nuclear delivery in cancer cells, and receptor-mediated uptake in triple-negative breast cancer models	(Aghaz et al., 2023; Fattahi et al., 2024; González-Urías et al., 2021; Huang et al., 2022; Newham et al., 2021; Xiao et al., 2021)
Penetration into three-dimensional and difficult tumor regions	Responsiveness designed to overcome spheroid, hypoxic, stromal, clonogenic, or poorly perfused tumor barriers	Three-dimensional MCF-7 spheroids, U87MG spheroids, three-dimensional tumorspheres, transparent tumor tissues, and tumor-microenvironment-on-a-chip models	Higher-order models showed whether platforms reached biologically resistant compartments. Liposomal nanoparticles failed to diffuse beyond 100 μm from vessels, whereas hypoxia-inducible factor 1 alpha-positive cells extended up to approximately 460 μm and cluster of differentiation 44-positive cells extended to approximately 300 μm. Other systems reduced U87MG spheroid formation, inhibited three-dimensional tumorsphere growth, or used drug-loaded macrophages to infiltrate tumor spheroids and increase cytotoxicity	(Mamnoon et al., 2020; Migasova et al., 2025; Samson et al., 2020; Tan et al., 2024; Wang et al., 2020)
*In vivo* tumor suppression	Stimulus-responsive release, targeting, or combination therapy translated into measurable tumor control	Murine xenograft, orthotopic, BALB/c, CT26, H22, and breast cancer tumor models; tumor-growth monitoring, biodistribution, and tumor accumulation	*In vivo* outcomes included 60.1% colorectal tumor-growth inhibition, 75.12% tumor inhibition in H22 tumor-bearing mice, approximately 100% tumor regression in BALB/c mice, 88.0% tumor inhibition for doxorubicin/B-cell lymphoma 2 small interfering RNA co-delivery, 87.29% tumor inhibition in CT26 tumors, and 76.98% ± 9.09% tumor suppression in hormonal mouse breast cancer models	(Fu et al., 2025; He et al., 2021; Li et al., 2024; Yan et al., 2020; Yan et al., 2023; Zhang et al., 2025b)
Metastasis, recurrence, survival, and treatment resistance	Responsive platforms evaluated against progression-related endpoints beyond primary tumor shrinkage	4T1 metastasis models, ovarian cancer recurrence/metastasis models, glioblastoma xenografts, resistant breast cancer models, and triple-negative breast cancer models	Advanced validation showed reduced metastatic burden, lower recurrence, or survival benefit. Reported outcomes included an 80% lower recurrence rate after tumor resection, reduced lung metastasis in 4T1 models, inhibition of ovarian cancer recurrence and metastasis, and median survival extension from 26 to 56 days in glioblastoma-bearing mice	(Chen et al., 2026; Hu et al., 2022; Song et al., 2023; Xiao et al., 2021; Zhang et al., 2025a; Zhang et al., 2022; Zhou et al., 2023)
Immune and stromal microenvironment remodeling	Platforms designed to activate antitumor immunity, repolarize macrophages, relieve stromal barriers, or remodel extracellular matrix	Orthotopic lung cancer, pancreatic cancer, ovarian cancer, CT26 tumors, and osteosarcoma models; immune-cell profiling, cytokines, macrophage polarization, and extracellular matrix remodeling	Chemoimmunotherapy and stromal-modulating systems induced immunogenic cell death, increased cluster of differentiation 8-positive T-cell infiltration, promoted dendritic-cell maturation, shifted macrophages from M2 toward M1 phenotypes, increased tumor necrosis factor alpha and interferon gamma, degraded collagen-rich extracellular matrix, and improved drug penetration and distribution	(Jiang et al., 2025; Li et al., 2025; Wang et al., 2025b; Yan et al., 2023; Zhang et al., 2023b; Zhang et al., 2024b; Zhang et al., 2025a)
Safety, selectivity, and tolerability	Precision benefit assessed by normal-cell sparing, hemocompatibility, reduced cardiotoxicity, or lower systemic toxicity	Human umbilical vein endothelial cells, HaCaT cells, L02 cells, L929 cells, 3T3 cells, peripheral blood mononuclear cells, and human foreskin fibroblast 2 cells; hemolysis, zebrafish embryo toxicity, organ toxicity, cardiotoxicity, and systemic toxicity	Selectivity data supported therapeutic precision when paired with tumor efficacy. Examples included minimal normal-cell uptake by aptamer-functionalized, doxorubicin-loaded pollen-like silica particles, approximately 100% human foreskin fibroblast 2 cell viability and <5% hemolysis for methotrexate/chloroquine nanohydrogels, zebrafish embryo median lethal dose values of 1,185.93 and 823.03 μM for ruthenium-loaded nanogels, and reduced cardiotoxicity or systemic toxicity in several *in vivo* systems	(Abd Al-Jabbar et al., 2022; Du et al., 2020; Jia et al., 2020; Miao et al., 2020; Pragti et al., 2023; Rashidzadeh et al., 2023; Shi et al., 2020; Wang et al., 2025a; Xiao et al., 2021; Zhang et al., 2025b)

## Translational bioengineering significance

### Stimuli-responsive control as the basis of precision cancer therapy

**FIGURE 5:**
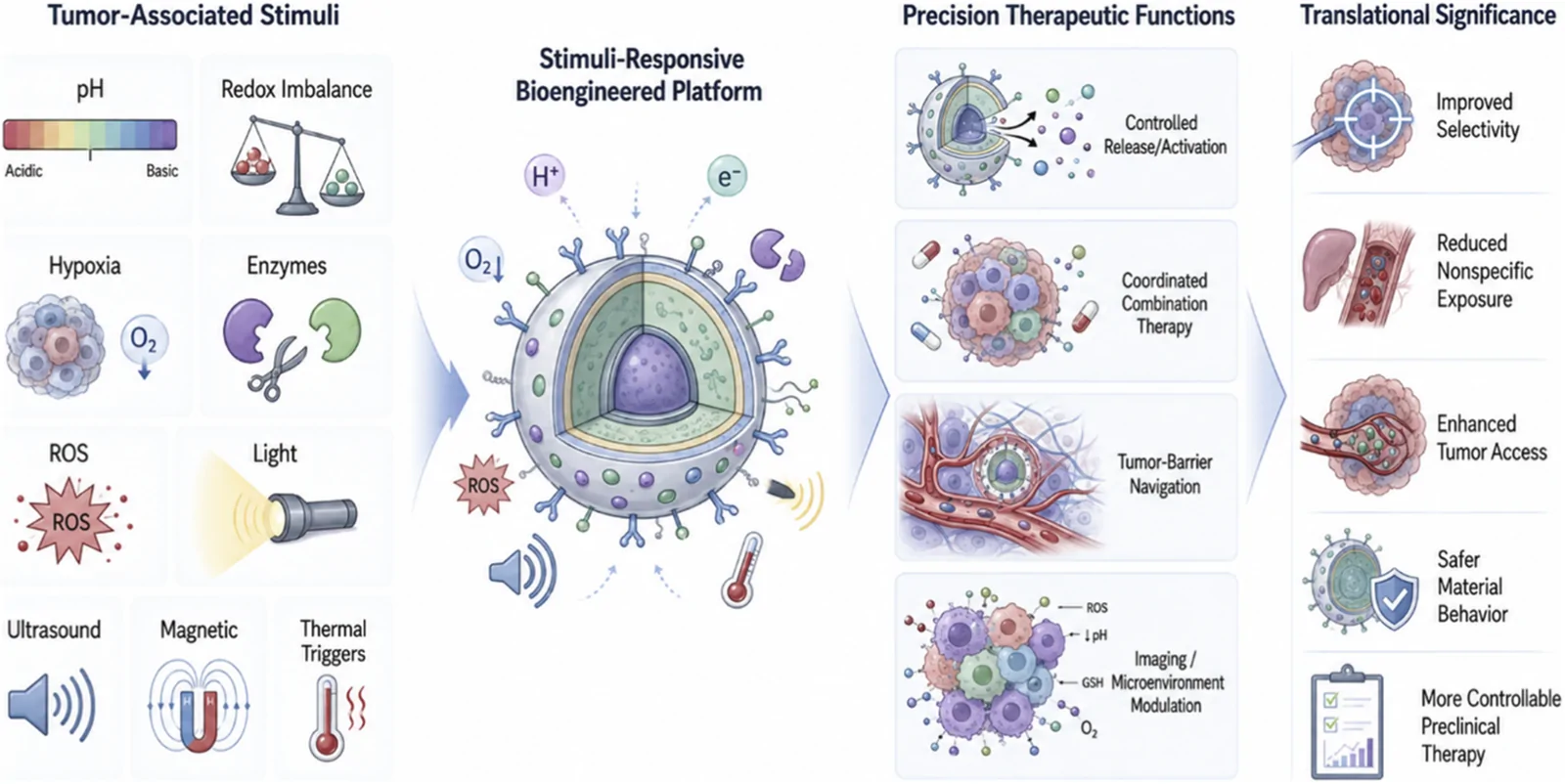
Translational Bioengineering Significance of Stimuli-Responsive Platforms in Precision Cancer Therapy. This figure demonstrates the translational logic of stimuli-responsive bioengineered platforms for precision cancer therapy. Tumor-associated biological cues, including acidic pH, redox imbalance, hypoxia, enzymatic activity, and ROS, together with exogenous triggers such as light, ultrasound, magnetic fields, and thermal stimuli, guide platform activation. The central bioengineered carrier integrates cue recognition with controlled release, therapeutic payload delivery, and reporter-based tracking. These engineered responses support precision delivery, coordinated combination therapy, tumor-barrier navigation, microenvironment modulation, improved selectivity, enhanced tumor access, reduced nonspecific exposure, and safer material behavior (Prepared with the assistance of Gemini’s NotebookLM Pro and ChatGPT Plus).

Stimuli-responsive bioengineered platforms represent a translational shift from conventional systemic drug administration toward therapeutic systems that are activated, remodeled, or released in response to tumor-specific conditions. Across the reviewed studies, precision is achieved by aligning material behavior with pathological cues, including acidic pH, redox imbalance, hypoxia, enzymatic activity, ROS, temperature variation, magnetic responsiveness, light activation, ultrasound stimulation, and sequential extracellular or intracellular triggers (Augustine et al., 2020; Gebrie et al., 2022; Guo et al., 2020; Jia et al., 2020; Li Y. C. et al., 2020; Mamnoon et al., 2020; Rashidzadeh et al., 2023; Tao and Yin, 2020; Wang L. et al., 2025; Wang et al., 2024a; Zhang D. et al., 2024; Zhang N. X. et al., 2021; Zhang Y. P. et al., 2025). This design logic is central to precision cancer therapy because therapeutic performance is not pursued only through drug potency but also through engineered control over when, where, and how a therapeutic payload becomes available. By maintaining stability under physiological conditions and responding preferentially within tumor-relevant environments, these systems provide a stronger basis for selective treatment than free-drug administration or nonresponsive carriers (Guo et al., 2020; Jia et al., 2020; Li and Liu, 2022; Miao et al., 2022; Shi et al., 2020; Tao and Yin, 2020; Xiao et al., 2021; Yang et al., 2020). The conceptual sequence connecting tumor-associated stimuli, platform responsiveness, therapeutic function, and translational value is summarized in Figure 5.

### From drug carriers to coordinated therapeutic systems

A defining translational contribution of these platforms is their ability to integrate more than one therapeutic or delivery function within a single engineered system. The reviewed studies include platforms designed for dual-drug chemotherapy, gene–drug co-delivery, chemoimmunotherapy, chemo-photothermal therapy, chemo-photodynamic therapy, chemodynamic therapy, ferroptosis-based treatment, radiosensitization, sonodynamic activation, and protein–photosensitizer co-delivery (Arvejeh et al., 2025; Gao et al., 2025; Liu et al., 2025; Phung et al., 2023; Song et al., 2023; Wang et al., 2026; Yan et al., 2020; Yan et al., 2023; Yang et al., 2022; Zhang F. et al., 2023; Zhang G. Z. et al., 2023; Zhang L. et al., 2024; Zhang L. et al., 2025; Zhang N. X. et al., 2021; Zhang et al., 2022; Zhang Y. P. et al., 2025; Zhang and Liang, 2024; Zhou et al., 2023). This multifunctionality is important because tumor heterogeneity, therapeutic resistance, immune suppression, recurrence, metastasis, and poor monotherapy response are rarely addressed effectively by single-agent treatment alone (Hu et al., 2022; Karimifard et al., 2022; Mathes et al., 2024; Song et al., 2023; Zhang F. et al., 2023; Zhang L. et al., 2025; Zhou et al., 2023). Stimuli-responsive platforms can synchronize or sequence complementary therapeutic actions, thereby supporting more rational combination-treatment design while limiting nonspecific exposure (Gao et al., 2025; Li et al., 2025; Song et al., 2023; Yan et al., 2023; Zhang G. Z. et al., 2023; Zhang L. et al., 2024; Zhang and Liang, 2024). In this context, their translational significance extends beyond cargo transport; they operate as programmable therapeutic systems that coordinate delivery, activation, and biological modulation within the tumor setting.

### Engineering around tumor barriers

The reviewed literature also shows that precision cancer therapy depends on overcoming biological barriers that limit passive delivery. One study directly demonstrates that standard liposomal nanoparticles may fail to reach hypoxic and clonogenic tumor regions beyond their diffusion limits, underscoring the need for delivery systems designed for deeper tumor access rather than reliance on passive accumulation alone (Samson et al., 2020). Other studies address this challenge through charge-reversal behavior, tumor-penetrating carriers, extracellular matrix or stromal modulation, transcytosis-oriented strategies, macrophage-mediated delivery, and tumor microenvironment remodeling (Jia et al., 2022; Jiang et al., 2025; Qian et al., 2020; Tang et al., 2021; Wang et al., 2020; Wang L. J. et al., 2025; Wang et al., 2024a; Wang et al., 2024b). These approaches are translationally relevant because they target practical barriers that commonly reduce therapeutic performance, including dense extracellular matrices, hypoxic niches, inadequate intracellular access, rapid clearance, protein interactions, and poor tissue penetration (He et al., 2021; Li et al., 2025; Samson et al., 2020; Tang et al., 2021; Wang et al., 2020; Wang L. J. et al., 2025; Wang et al., 2024a; Wang et al., 2024b). By treating these barriers as engineering constraints rather than secondary biological complications, stimuli-responsive platforms more directly support the goals of precision oncology.

### Safety, practicality, and translational fit

For stimuli-responsive systems to be translationally meaningful, responsiveness must be paired with material safety, reproducibility, and practical development potential. Several studies therefore emphasize biocompatible, biodegradable, hemocompatible, naturally derived, green-synthesized, modular, simplified, or excretable systems (Christodoulou et al., 2025; Dominski et al., 2022; Li et al., 2024; Miao et al., 2020; Newham et al., 2021; Nie et al., 2023; Qian et al., 2020; Sasikala et al., 2025; Xu et al., 2022; Yang et al., 2020; Ye et al., 2020). These features are especially important for polymeric, silica-based, protein-derived, cellulose-based, and hybrid inorganic–organic platforms, where therapeutic performance must be balanced against premature leakage, long-term retention, systemic toxicity, fabrication complexity, and formulation practicality (Li et al., 2024; Newham et al., 2021; Nie et al., 2023; Raj and Sundaramoorthy, 2025; Xiao et al., 2021; Xu et al., 2022; Yang et al., 2020; Ye et al., 2020). The most translationally relevant platforms are therefore not simply the most complex or multifunctional; rather, they combine controlled therapeutic behavior with plausible safety, manufacturability, reproducibility, and material-fate profiles. This distinction helps clarify the translational gap addressed by the field: stimulus responsiveness must be engineered not only for biological precision but also for development feasibility.

### Monitorable and microenvironment-modulating platforms

A further translational pattern is the emergence of platforms that combine therapy with tracking, imaging, or tumor microenvironment modulation. Some systems integrate fluorescence, bioimaging, computed tomography capability, or other tracking functions, extending precision therapy from controlled delivery to monitorable treatment (González-Urías et al., 2021; Mahani et al., 2025; Maiti et al., 2025; Namazi et al., 2023; Perera et al., 2022; Zhang G. Z. et al., 2023). Others actively remodel tumor acidity, hypoxia, immune suppression, macrophage phenotype, stromal structure, extracellular matrix density, or vascular and tissue barriers (Gao et al., 2025; Jiang et al., 2025; Li et al., 2025; Qian et al., 2020; Song et al., 2023; Tang et al., 2021; Wang L. J. et al., 2025; Yan et al., 2023; Zhang G. Z. et al., 2023; Zhang L. et al., 2024; Zhang L. et al., 2025). This expands the role of stimuli-responsive biomaterials from passive delivery vehicles into systems capable of modifying the tumor ecosystem to improve therapeutic responsiveness. The significance of these approaches is their ability to connect localized therapeutic action with the broader biological context that shapes cancer progression and treatment failure.

### Overall translational perspective

Taken together, the reviewed studies support the continued development of stimuli-responsive bioengineered platforms as precision-oriented preclinical strategies for cancer therapy. Their significance lies in demonstrating how biomaterials can integrate tumor targeting, microenvironment sensing, controlled release, multimodal treatment, immune or stromal modulation, imaging-enabled tracking, and resistance management within adaptable delivery technologies (González-Urías et al., 2021; Mahani et al., 2025; Namazi et al., 2023; Perera et al., 2022; Tang et al., 2021; Wang et al., 2024b; Zhang G. Z. et al., 2023). The broader translational value of this field is therefore not limited to improving drug loading or circulation time; rather, it lies in engineering therapeutic systems that respond to the biological complexity of tumors with greater spatial, temporal, and functional control. Within the framework of precision cancer therapy, these platforms provide a foundation for safer, more selective, and more coordinated treatment strategies where conventional drug administration is limited by systemic toxicity, poor selectivity, inadequate tumor penetration, or insufficient control over combination therapy. Table 5 summarizes the major translational design patterns that support this precision-oriented framework.

**TABLE 5:** Translational bioengineering significance of stimuli-responsive platforms in precision cancer therapy.

Translational bioengineering significance	Stimuli-responsive platform logic	Precision cancer therapy value	Most appropriate evidence emphasis for this section	Representative references
Tumor-adaptive therapeutic activation	Platforms are engineered to respond to tumor-associated cues, including acidic pH, redox imbalance, hypoxia, enzymes, ROS, temperature, ultrasound, light, magnetic guidance, or sequential extracellular/intracellular triggers	Enables therapeutic release or activation to occur preferentially within tumor-relevant environments rather than during systemic circulation	Emphasize tumor-triggered control, selective activation, and reduced nonspecific exposure; avoid detailed release percentages unless essential to the translational argument	(Augustine et al., 2020; Gebrie et al., 2022; Guo et al., 2020; Jia et al., 2020; Mamnoon et al., 2020; Tao and Yin, 2020; Wang et al., 2024a; Zhang et al., 2024a; Zhang et al., 2021b; Zhang et al., 2025b)
Integrated combination-treatment design	Single platforms coordinate multiple therapeutic functions, including dual-drug delivery, gene–drug therapy, chemoimmunotherapy, chemo-phototherapy, ferroptosis, radiosensitization, sonodynamic therapy, or protein/photosensitizer co-delivery	Supports precision therapy by synchronizing or sequencing complementary mechanisms against resistance, recurrence, metastasis, immune suppression, or poor monotherapy response	Emphasize the translational rationale for coordinated therapy rather than individual assay outcomes or detailed mechanisms	(Arvejeh et al., 2025; Gao et al., 2025; Liu et al., 2025; Phung et al., 2023; Song et al., 2023; Wang et al., 2026; Yan et al., 2020; Yan et al., 2023; Yang et al., 2022; Zhang et al., 2023a; Zhang et al., 2023b; Zhang et al., 2024b; Zhang et al., 2025a; Zhang et al., 2021b; Zhang et al., 2022; Zhang et al., 2025b)
Engineering around delivery barriers	Responsive systems are designed to address poor tumor penetration, dense stroma, extracellular matrix barriers, hypoxia, protein interactions, rapid clearance, and inadequate intracellular access	Reframes tumor barriers as engineering targets, improving the likelihood that therapeutic payloads reach difficult tumor regions	Include barrier-defining evidence where it directly supports translational need, such as limited penetration of standard liposomal nanoparticles into hypoxic or clonogenic tumor regions	(Jia et al., 2022; Jiang et al., 2025; Qian et al., 2020; Samson et al., 2020; Shi et al., 2020; Tang et al., 2021; Wang et al., 2020; Wang et al., 2025b; Wang et al., 2024a; Wang et al., 2024b; Zhang et al., 2021a)
Material safety and translational practicality	Platforms incorporate biocompatible, biodegradable, hemocompatible, naturally derived, green-synthesized, modular, simplified, or excretable materials	Links therapeutic responsiveness with safety, manufacturability, reproducibility, and reduced long-term material burden	Emphasize biodegradation, excretion, simplified synthesis, reduced toxicity, practical formulation logic, and development feasibility; reserve detailed physicochemical values for formulation-focused sections	(Christodoulou et al., 2025; Li et al., 2024; Miao et al., 2020; Newham et al., 2021; Nie et al., 2023; Qian et al., 2020; Raj and Sundaramoorthy, 2025; Sasikala et al., 2025; Xu et al., 2022; Yang et al., 2020; Ye et al., 2020)
Theranostic and trackable precision platforms	Some systems combine therapeutic delivery with fluorescence, bioimaging, computed tomography capability, or other tracking functions	Extends precision therapy from controlled delivery to monitorable treatment, allowing platform localization or therapeutic tracking	Emphasize integration of treatment and tracking; avoid duplicating imaging methodology unless needed in a dedicated theranostic subsection	(González-Urías et al., 2021; Mahani et al., 2025; Maiti et al., 2025; Namazi et al., 2023; Perera et al., 2022; Zhang et al., 2023b)
Tumor microenvironment modulation	Certain platforms actively remodel acidity, hypoxia, immune suppression, macrophage phenotype, stromal structure, extracellular matrix density, or vascular/tissue barriers	Expands stimuli-responsive biomaterials from passive delivery vehicles into systems that modify the tumor ecosystem to improve treatment responsiveness	Emphasize microenvironment-level therapeutic significance rather than detailed pathway descriptions, which belong in mechanism-focused sections	(Gao et al., 2025; Jiang et al., 2025; Li et al., 2025; Qian et al., 2020; Song et al., 2023; Tang et al., 2021; Wang et al., 2025b; Yan et al., 2023; Zhang et al., 2023b; Zhang et al., 2024b; Zhang et al., 2025a)

## Limitations and future perspectives

Despite strong preclinical momentum, several limitations constrain the translational maturity of stimuli-responsive bioengineered cancer platforms. First, much of the current evidence remains concentrated in early-stage experimental systems. Cellular assays, uptake studies, cytotoxicity testing, apoptosis analysis, and small-animal tumor models provide important mechanistic and therapeutic signals, but they do not fully capture patient-level heterogeneity, human tumor architecture, immune diversity, pharmacokinetic variability, or long-term material fate. Studies incorporating spheroids, transparent tumor tissues, tumor-microenvironment-on-a-chip models, and orthotopic or metastatic models represent important progress, yet these approaches are still inconsistently applied across the field (Mamnoon et al., 2020; Migasova et al., 2025; Samson et al., 2020; Tan et al., 2024; Wang et al., 2020; Wang L. J. et al., 2025).

Second, stimulus responsiveness is often demonstrated under simplified experimental conditions that may not reproduce the gradients, timing, intensity, and heterogeneity of human tumors. Acidic pH, elevated GSH, hypoxia, enzyme abundance, ROS, or externally applied energy can vary substantially across tumor types, disease stages, and even regions within the same lesion. Future studies should therefore quantify stimulus thresholds, activation kinetics, and release behavior under biologically realistic conditions, including heterogeneous three-dimensional models and patient-derived systems. Platforms should be evaluated not only for whether they respond, but also for whether their response occurs within clinically meaningful ranges and produces sufficient therapeutic gain over nonresponsive controls (Gupta et al., 2022; Hu et al., 2022; Li et al., 2021; Mamnoon et al., 2020; Rashidzadeh et al., 2023; Tao and Yin, 2020; Yang et al., 2020; Zhang Y. P. et al., 2025).

Third, the complexity that enables multifunctionality may also hinder translation. Many advanced systems combine multiple materials, cargos, coatings, targeting ligands, cleavable linkers, inorganic components, imaging elements, and externally activated modules. Although such designs can produce impressive preclinical performance, they may introduce barriers related to batch reproducibility, scale-up, sterilization, stability, quality control, regulatory characterization, and cost. Future development should prioritize rational simplification: platforms should retain only those functions that produce measurable therapeutic advantage. Biocompatible, biodegradable, hemocompatible, naturally derived, green-synthesized, self-assembled, modular, and excretable systems are particularly attractive when they combine mechanistic precision with practical manufacturability (Christodoulou et al., 2025; Dominski et al., 2022; Li et al., 2024; Miao et al., 2020; Newham et al., 2021; Nie et al., 2023; Qian et al., 2020; Raj and Sundaramoorthy, 2025; Sasikala et al., 2025; Xu et al., 2022; Yang et al., 2020; Ye et al., 2020).

Fourth, safety evaluation remains uneven. Many studies include normal-cell controls, hemolysis assays, zebrafish testing, cardiotoxicity monitoring, or organ-toxicity observations, but long-term biodistribution, degradation products, immunogenicity, repeated dosing, clearance routes, and chronic toxicity are less consistently addressed. This gap is especially important for inorganic, hybrid, metal-containing, silica-based, magnetic, and framework-based platforms, where material persistence and biological transformation may influence clinical acceptability. Future studies should integrate efficacy, pharmacokinetics, toxicology, immunological profiling, and material-fate assessment into a unified development framework rather than treating safety as a late-stage confirmation (Jia et al., 2020; Miao et al., 2020; Shi et al., 2020; Wang L. et al., 2025; Xiao et al., 2021; Yang et al., 2020; Zhang D. et al., 2024; Zhang Y. P. et al., 2025).

Fifth, tumor penetration remains a fundamental unresolved challenge. Evidence that conventional liposomal nanoparticles may not reach hypoxic and clonogenic tumor regions beyond limited perivascular distances highlights the need to evaluate spatial drug distribution, not merely total tumor accumulation (Samson et al., 2020). Future platforms should be tested for penetration into stromal, hypoxic, necrotic, immune-suppressed, and therapy-resistant compartments. Designs based on charge conversion, size reduction, matrix remodeling, transcytosis, cell-mediated transport, and microenvironment normalization are promising, but they require rigorous comparison against clinically relevant standards and spatially resolved validation methods (Jia et al., 2022; Qian et al., 2020; Samson et al., 2020; Shi et al., 2020; Tan et al., 2024; Wang et al., 2020; Wang L. J. et al., 2025; Wang et al., 2024a; Wang et al., 2024b).

Sixth, multimodal and co-delivery systems require stronger evidence of mechanistic necessity. Combination platforms often show superior efficacy, but future studies should clarify whether each component contributes independently, synergistically, or redundantly. Dose ratios, release sequence, intracellular localization, immune activation, resistance reversal, and toxicity mitigation should be quantitatively connected to therapeutic outcomes. This is particularly important for platforms addressing multidrug resistance, gene modulation, chemoimmunotherapy, radiosensitization, recurrence, metastasis, or multilevel resistance mechanisms (Gao et al., 2025; Li et al., 2025; Liu et al., 2025; Phung et al., 2023; Song et al., 2023; Yan et al., 2020; Yan et al., 2023; Zhang F. et al., 2023; Zhang L. et al., 2025; Zhang et al., 2022; Zhou et al., 2023; Puzzo et al., 2025; Puzzo et al., 2024).

Finally, the field should move toward biomarker-guided platform selection. Not every tumor will benefit from the same trigger, architecture, or delivery strategy. Tumors dominated by hypoxia may require hypoxia-responsive or oxygen-modulating systems; matrix-rich tumors may require stromal remodeling or enzyme-responsive penetration strategies; resistant tumors may require gene–drug co-delivery, chemosensitization, or immune modulation; and tumors accessible to external energy may benefit from light-, ultrasound-, or magnetically assisted activation. Future studies should therefore link platform design to measurable tumor phenotypes, enabling a more clinically actionable form of precision bioengineering. Data-driven formulation tools, imaging-enabled platforms, and monitorable theranostic systems may accelerate this transition by connecting material design, tumor biology, and therapeutic response within an integrated translational workflow (González-Urías et al., 2021; Gorish et al., 2026; Mahani et al., 2025; Maiti et al., 2025; Namazi et al., 2023; Perera et al., 2022; Tang et al., 2021; Wang et al., 2024b; Zhang G. Z. et al., 2023).

## Conclusion

Stimuli-responsive bioengineered platforms represent a major conceptual advance in precision cancer therapy because they transform drug delivery from a passive transport problem into a programmable therapeutic strategy. Across diverse material architectures and tumor models, these systems demonstrate how biological and externally applied cues can be used to regulate release, activation, penetration, multimodal treatment, immune or stromal modulation, and safety. Their promise lies in aligning therapeutic action with the spatial, temporal, and biological complexity of malignant tissue while reducing unnecessary exposure outside the tumor setting. The field’s next stage will depend on moving beyond increasingly complex prototypes toward clinically disciplined systems that are mechanistically justified, manufacturable, safe, spatially validated, and matched to defined tumor phenotypes. If these challenges are met, stimuli-responsive bioengineering could provide a powerful foundation for cancer therapies that are not only more targeted but also more adaptive, coordinated, and biologically precise.

The reviewed evidence supports a clear conceptual trajectory: stimuli-responsive bioengineered platforms have progressed from a rationale based on tumor-selective activation toward increasingly sophisticated preclinical systems that integrate controlled release, tumor penetration, multimodal therapy, microenvironment modulation, and safety-oriented design. However, the field remains largely preclinical, and its clinical value will depend on whether mechanistic responsiveness can be translated into reproducible, scalable, biomarker-guided, and clinically benchmarked therapeutic strategies. Table 6 synthesizes the evidence-to-practice pathway by distinguishing what is currently supported, what remains unresolved, and which priorities should guide the next stage of development.

**TABLE 6:** Evidence-to-practice roadmap for stimuli-responsive bioengineered platforms in precision cancer therapy.

Roadmap stage	What we know	What we do not yet know	Practice-facing interpretation	Future priorities
Rationale: why responsive platforms matter	Tumor-associated cues can be used to improve when, where, and under which biological conditions therapy becomes active. The central rationale is not drug carriage alone but spatial, temporal, and biological control of therapeutic action	It remains unclear which tumor cues are sufficiently strong, stable, and patient-specific to serve as reliable clinical triggers across heterogeneous tumors	Stimuli-responsive design is most compelling when it solves a defined therapeutic problem, such as poor selectivity, premature exposure, inadequate intracellular release, resistance, or inaccessible tumor regions	Define tumor-cue thresholds that are clinically actionable; match platform design to measurable tumor phenotypes rather than assuming universal responsiveness
Mechanism: how the platform becomes active	Responsive systems can convert pH, redox imbalance, hypoxia, ROS, enzymes, temperature, light, ultrasound, or related cues into material transitions such as release, degradation, swelling, charge conversion, disassembly, deshielding, or externally triggered activation	Many studies demonstrate responsiveness under simplified experimental conditions, but the relationship among stimulus intensity, activation kinetics, intratumoral heterogeneity, and therapeutic benefit is not yet standardized	Mechanistic claims are strongest when carrier activation is quantitatively linked to controlled release, leakage suppression, penetration, intracellular access, or therapeutic response	Require stimulus-threshold testing, kinetic release profiling, leakage controls, nonresponsive comparators, and biologically realistic models that reproduce tumor gradients
Platform design: what engineering contributes	Material architecture determines whether responsiveness is useful: the carrier must protect cargo during circulation, remain stable before activation, and transition predictably under tumor-relevant conditions	It remains uncertain which architectures offer the best balance among responsiveness, simplicity, reproducibility, scalability, safety, and material clearance	The most practice-relevant platforms will not necessarily be the most complex; they will be those that combine controlled activation with manufacturable and biologically acceptable design	Prioritize rationally simplified architectures, scalable synthesis, batch-to-batch reproducibility, defined critical quality attributes, and early material-fate assessment
Preclinical testing: what has been validated	Evidence is strongest in cell-based assays, uptake studies, cytotoxicity testing, apoptosis assays, spheroids, tumor-bearing animal models, and selected endpoints such as tumor suppression, metastasis reduction, recurrence control, survival extension, immune activation, stromal remodeling, and reduced off-target toxicity	Evidence remains uneven across tumor types, models, dosing schedules, long-term safety, biodistribution, immune effects, and comparisons against clinically relevant standards	Current evidence supports strong preclinical plausibility, but not yet broad clinical readiness. Responsive behavior must be validated as a therapeutic advantage, not only as a material property	Use standardized validation pipelines, orthotopic and metastatic models, patient-derived systems, spatial penetration assays, long-term toxicity studies, and clinically relevant comparator arms
Populations and tumor contexts: who may benefit	The reviewed platforms address diverse solid tumors, with particular relevance to tumors limited by poor penetration, hypoxia, dense stroma, immunosuppression, recurrence, metastasis, or resistance	The field has not yet established which patients, tumor phenotypes, or disease stages are most likely to benefit from specific responsive designs	Clinical translation should move from “one responsive platform for cancer” toward phenotype-matched therapy, in which the platform is selected according to tumor biology	Develop biomarker-guided selection criteria based on acidity, redox state, hypoxia, enzyme activity, stromal density, immune phenotype, vascular access, and resistance profile
Outcomes: what benefit should be measured	Meaningful outcomes extend beyond short-term cytotoxicity and include tumor penetration, intracellular delivery, apoptosis, tumor-growth inhibition, metastasis, recurrence, survival, immune remodeling, stromal modulation, hemocompatibility, cardiotoxicity, and systemic safety	There is no uniform hierarchy of endpoints for judging whether a responsive platform is superior to a free drug, nonresponsive carrier, or standard treatment	Practice-facing value depends on demonstrating an improved therapeutic index: greater tumor control with lower off-target exposure or toxicity	Establish outcome tiers: mechanism confirmation, delivery advantage, biological effect, therapeutic superiority, safety advantage, and translational feasibility
Clinical-practice pathway: what is closest to implementation	Platforms with simple composition, strong leakage control, clear activation logic, biocompatible or biodegradable components, monitorable behavior, and compatibility with existing therapeutic regimens appear most development-ready	Regulatory feasibility, large-scale manufacturing, sterilization, storage stability, repeat dosing, immune compatibility, and human pharmacokinetics remain insufficiently resolved	The immediate clinical opportunity is not wholesale replacement of cancer therapy but improved delivery, localization, coordination, or sensitization of established therapeutic modalities	Focus on clinically realistic use cases: localized delivery, adjuvant therapy, resistant tumors, tumor-penetration enhancement, combination-therapy optimization, and monitorable treatment response
Future gap: what must change before practice adoption	The field has generated strong proof-of-concept evidence that tumor biology can be engineered as a trigger for precision therapy	The major gap is the absence of a standardized evidence chain connecting stimulus responsiveness to manufacturable design, validated tumor targeting, clinically relevant efficacy, long-term safety, and patient selection	Stimuli-responsive platforms should now be evaluated as translational therapeutic systems, not as isolated nanocarrier innovations	Build an evidence-to-practice pipeline: clinically relevant stimulus mapping, simplified platform design, standardized preclinical benchmarks, patient-derived validation, spatial biodistribution analysis, long-term toxicology, scalable manufacturing, and biomarker-guided trial design
